# Functional characterization of the polar organizer protein FimV in *Pseudomonas putida*

**DOI:** 10.1128/jb.00497-25

**Published:** 2026-01-12

**Authors:** Lisa Marie Schmidt, Marta Pulido-Sánchez, Anke Treuner-Lange, Lukas Zehner, Aroa López-Sánchez, Felipe Cava, Fernando Govantes, Kai M. Thormann

**Affiliations:** 1 Institute of Microbiology and Molecular Biology, Justus-Liebig-Universität Gießen 9175, Gießen, Germany; 2 Centro Andaluz de Biologia del Desarrollo, Universidad Pablo de Olavide/Consejo Superior de Investigaciones Cientificas/Junta de Andalucia 85097, Seville, Spain; 3 Departamento de Biologia Molecular e Ingenieria Bioquimica, Universidad Pablo de Olavide 16772, Seville, Spain; 4 Max Planck Institute for Terrestrial Microbiology 28310, Marburg, Germany; 5 Department of Molecular Biology, The Laboratory for Molecular Infection Medicine Sweden (MIMS), Umeå Center for Microbial Research (UCMR), Science for Life Laboratory (SciLifeLab), Umeå University 8075, Umeå, Sweden; Geisel School of Medicine at Dartmouth, Hanover, New Hampshire, USA

**Keywords:** cellular asymmetry, HubP, landmark, polarity

## Abstract

Homologs of the polar landmark proteins HubP and FimV are widespread among bacterial species. They all share several common features, including a periplasmic LysM-like domain, a transmembrane region, an extensive cytoplasmic domain enriched in acidic amino acids, and a C-terminal tetrapeptid-repeat (TPR) domain referred to as the FimV domain. Apart from these conserved general features, however, the proteins exhibit little homology across different bacterial genera. Here, we characterized *Pseudomonas putida* FimV (*Pp*FimV) with respect to cellular processes involving FimV or HubP in other species. We found that *Pp*FimV nonspecifically binds to peptidoglycan via its periplasmic LysM domain, which, together with an immunoglobulin-like domain, is necessary for proper polar positioning. *Pp*FimV is required for normal flagellar-mediated swimming and the placement of the chemotaxis system. However, *Pp*FimV is not involved in regulating the number of flagellar filaments, chromosome segregation, or type IV pilus-dependent surface motility. Thus, *Pp*FimV has surprisingly little functional overlap with, for example, HubP from *Vibrio* sp. or *Shewanella putrefaciens* or with FimV from *P. aeruginosa. Pp*FimV was unable to compensate for the loss of *Sp*HubP with regard to swimming in soft agar, and *vice versa*. Domain swapping between *Sp*HubP and *Pp*FimV revealed that differences in the cytoplasmic region between the transmembrane region and the C-terminal FimV domain likely account for the proteins’ distinct functions in flagella-mediated swimming. This suggests that FimV and HubP are structural homologs that have evolved to perform different, species-specific functions.

**IMPORTANCE:**

Many bacterial species possess landmark proteins that organize the bacterial cell and localize specific cellular processes to the cell’s polar regions by directing client proteins or protein complexes to their designated positions. FimV and its homolog HubP are landmark proteins found in many species of the gammaproteobacteria, but their roles are not well understood. Here, we demonstrate that only certain functions related to flagella-mediated motility appear to be conserved between *Pseudomonas putida* FimV and *Shewanella putrefaciens* HubP. This finding suggests a significant degree of functional diversity.

## INTRODUCTION

Like their eukaryotic counterparts, bacterial cells exhibit a high degree of organization. This organization is crucial for proper cellular function, including chromosome organization and segregation, cell growth and differentiation, metabolism, regulation, and motility. In rod-shaped cells, a significant proportion of molecules and processes are localized to the cell’s polar regions. A notable characteristic of bacteria is their frequent asymmetry between the “old” and “new” poles, with the latter forming during cell division and fission. Several protein complexes are specifically recruited to a single pole. Examples include multicomplexes of motility systems, such as type IV pili, flagella, and chemotaxis arrays. Additionally, protein positions may dynamically alternate between poles during cell development or directional switches in twitching motility. Polar localization of proteins often depends on a diffusion-capture mechanism involving interactions with polar landmark proteins that designate position (1, 2).

**Fig 1:**
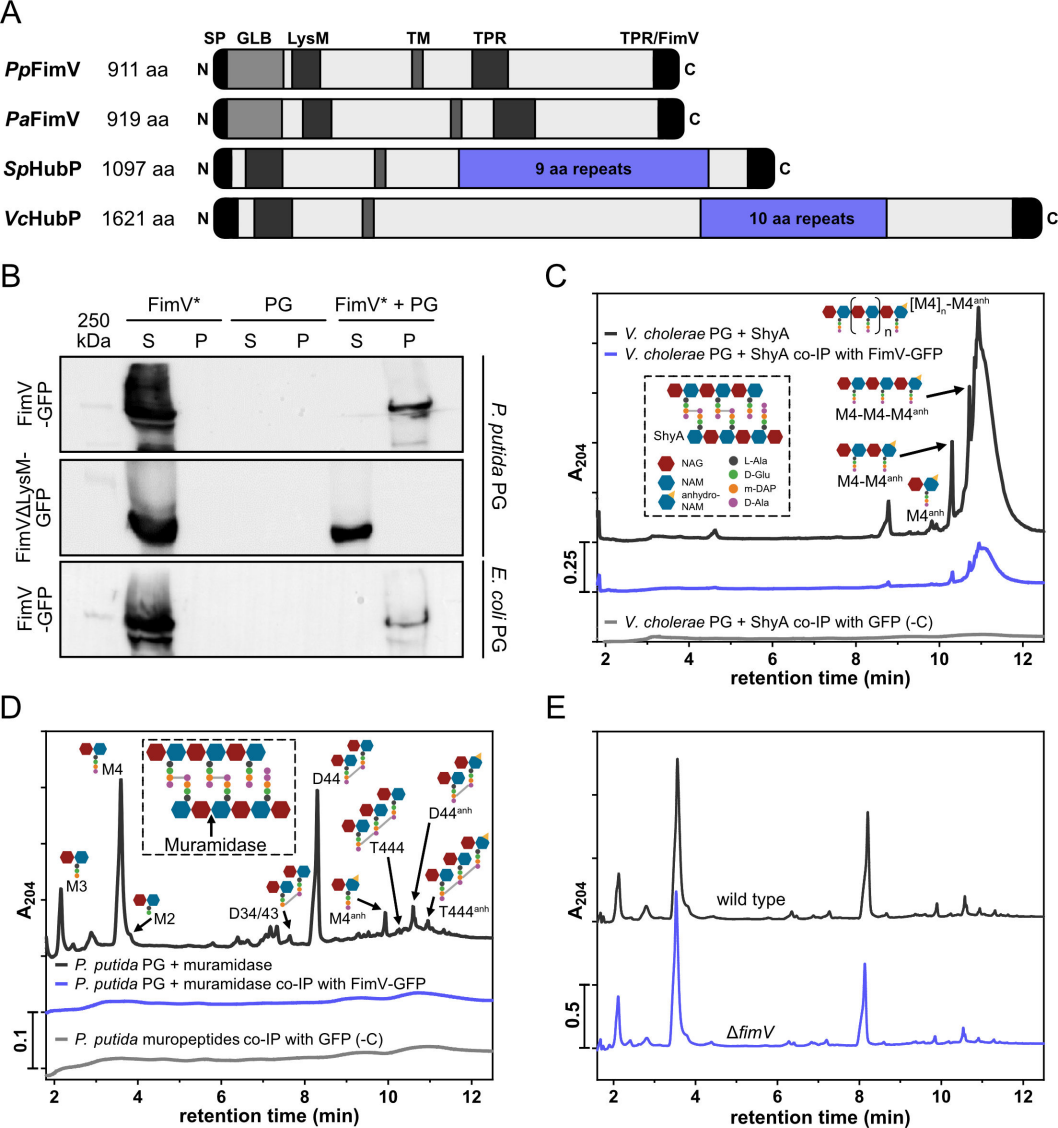
The periplasmic LysM domain of FimV interacts with the peptidoglycan cell wall. (**A**) Schematic overview of FimV proteins of *P. aeruginosa* and *P. putida* or HubP proteins of *S. putrefaciens* and *V. cholerae*, respectively. Protein sizes (amino acid [aa] numbers) and N- and C-terminal orientation are indicated. The following abbreviations for protein domains are used: signal peptide (SP), immunoglobulin-like domain (GLB), LysM-like domain (LysM), transmembrane region (TM), and tetratricopeptide repeat-like domain (TPR/FimV). (**B**) Western blot analysis of PG binding assays incubating purified FimV-GFP or FimVΔLysM-GFP with isolated sacculi from *P. putida* or *Escherichia coli* DH5α cells. Blots were probed with anti-GFP antibody. S: soluble fraction; P: pellet fraction; FimV*: purified FimV-GFP or FimVΔLysM-GFP; and PG: peptidoglycan. (**C**) ultra-performance liquid chromatography (UPLC) chromatograms showing soluble PG fragments released by *V. cholerae* O1 sacculus digestion with *Vc*ShyA, and the fragments co-immunoprecipitated with FimV-GFP and GFP (-C). The cartoon depicts the structure and components of the PG. An arrow denotes *Vc*ShyA cleavage. NAG: N-acetylglucosamine; NAM: N-acetylmuramic acid; and m-DAP: meso-diaminopimelic acid. (**D**) UPLC chromatograms showing soluble PG fragments released by *P. putida* sacculus digestion with muramidase, and the fragments co-immunoprecipitated with FimV-GFP and GFP (-C). An arrow denotes muramidase cleavage. (**E**) UPLC chromatograms showing the muropeptide profiles of wild-type *P. putida* and the Δ*fimV* mutant.

HubP, the “hub-of-the-pole” protein, is a major landmark protein that has been characterized in several gamma-proteobacteria, particularly in *Vibrio* species and *Shewanella putrefaciens* (3, 4). HubP is a transmembrane protein with an N-terminal periplasmic region that contains a LysM-like domain. The cytoplasmic regions of the HubP proteins in *Vibrio cholerae* (*Vc*HubP) and *S. putrefaciens* (*Sp*HubP) are largely unstructured and contain several copies of an imperfect repeat enriched in acidic amino acids (3, 4). At the C-terminus, HubP has a tetratricopeptide (TPR) domain that is thought to be the main binding interface for various client proteins (3–6). This domain is called the FimV domain due to apparent homologies to the C-terminal region of the *Pseudomonas aeruginosa* protein FimV (7) (see below). HubP orthologs are similar in general composition (N-terminal LysM-like domain, transmembrane region, acidic repeats, and C-terminal FimV domain) but surprisingly divergent at the amino acid level (3, 4). This low conservation particularly applies to the unstructured cytoplasmic region, which varies considerably in size among different HubP homologs. Additionally, the composition, length, and number of acidic repeats vary markedly between the cells of different genera (e.g., *Vibrio* and *Shewanella*) and between closely related species (e.g., *S. putrefaciens* and *Shewanella oneidensis* (4) (see Fig. 1A).

One of HubP’s defining characteristics is its intricate localization dynamics throughout the cell cycle. HubP forms a large cluster at the old flagellar cell pole and a small cluster at the new pole (3, 4). During cell growth and the onset of division, the small HubP cluster grows in size. Upon cell division, some HubP proteins localize to the division plane (3, 4). After cell fission is complete, the proteins reform the small cluster at the new cell pole. This asymmetric distribution of HubP likely contributes to polar identity in the corresponding species (3–5).

Studies have revealed that HubP has some conserved and some species-specific functions in *Vibrio* sp. and *S. putrefaciens*. In both species, HubP is involved in the segregation of the chromosome (chromosome 1, but not chromosome 2, in *V. cholerae*), by localizing *oriC* to the cell pole (3, 4, 8). HubP is also required for the proper localization of the chemotaxis multiprotein complex to the flagellated cell pole (3, 4, 9–11). Other identified HubP client proteins are the flagellar regulators FlhF and FlhG, which regulate the number and position of the polar flagellum, and the flagellar motor effector ZomB/MotV, which is necessary for the directional switching of the flagellar motor in *Vibrio* and *Shewanella* (3, 4, 12–14).

Other HubP clients appear to be more species-specific. For example, MotW and DacB are specific to *V. cholerae* (14). In *V. vulnificus*, HubP recruits the flagellar assembly effector FapA to the cell pole, linking flagellar formation to environmental glucose concentrations (15). In *S. putrefaciens*, HubP sequesters the multidomain phosphodiesterase PdeB (6). PdeB is only active when it interacts directly with the FimV domain of HubP. This interaction establishes asymmetry in cell division concerning the second messenger c-di-GMP, as well as heterogeneity in cellular behavior within the population (5, 6). These examples illustrate that, although not essential, HubP orchestrates numerous processes at the cell pole. It is likely that more HubP-dependent client proteins exist that provide general or species-specific polar functions to the cell.

Potential homologs of HubP can be found in numerous species of proteobacteria (16). In *P. aeruginosa*, the FimV protein (7) shares many key features with HubP. FimV has a periplasmic LysM-like domain, a cytoplasmic region, and the C-terminal FimV TPR domain, named after the FimV protein. Unlike HubP, FimV has no apparent repeat regions, but it does have an enrichment of acidic amino acids in the cytoplasmic section between the transmembrane and FimV domains (7). *P. aeruginosa* FimV (*Pa*FimV) was identified through transposon mutagenesis as an effector of type IV pili-mediated twitching in this species years before HubP was first described in *V. cholerae* (7). Further studies have provided evidence supporting a critical role for FimV in organizing the cell pole in *Pseudomonas*, particularly in localizing the type IV pilus apparatus and regulating its activity (17–19). Additionally, FimV interacts with regulatory components of the flagellar system (20–23) and mediates the levels of second messengers, such as cAMP and c-di-GMP (16, 19, 24).

Similar to HubP, FimV exhibits an intricate localization pattern and is required for establishing asymmetric cell division (23, 25). Therefore, we asked if other key roles are conserved between HubP and FimV. To this end, we compared the function of FimV in *P. putida* (*Pp*FimV) to that of *S. putrefaciens* HubP. While we confirmed the role of *Pp*FimV in flagellation, chemotaxis, and motility, we found that, unlike HubP in *Vibrio* and *Shewanella*, *Pp*FimV does not participate in chromosome segregation and is likely not required for type IV pili-mediated surface motility. Interestingly, swapping *S. putrefaciens* HubP domains with their *P. putida* FimV counterparts partially restored swimming phenotypes. The results demonstrate how the landmark proteins FimV and HubP have diverged in function between bacterial genera and species.

## RESULTS

### Properties of *Pp*FimV

Previous studies have identified and characterized FimV (PP_1993, *Pp*FimV) of *P. putida* concerning its localization pattern and role in the localization and polarity of its lophotrichous flagellar system (23, 26). The bioinformatic analysis (see Materials and Methods section) revealed that *Pp*FimV has 911 amino acid residues and a predicted molecular mass of 96.9 kDa and, thus, is similar in size to its *P. aeruginosa* counterpart (*Pa*FimV; 919 amino acids; Fig. 1A). The N-terminal signal peptide (SP; aa positions 1–24) is followed by a periplasmic region (aa 25–387) that contains a predicted LysM-like domain (aa 152–206). After a single transmembrane region (aa 388–408), *Pp*FimV has a 503-aa-residue-long cytoplasmic region with the hallmark TPR motif at the very C-terminus (aa 863–910). This general domain organization is similar to that of *Pa*FimV, HubP from *S. putrefaciens* (*Sp*HubP; (4)), and HubP from *V. cholerae* (*Vc*HubP; (3)). However, *Pp*FimV and *Vibrio* and *Shewanella* HubP proteins differ in several ways. The latter possesses several copies of an imperfect repeat with a high content of acidic aa residues (3, 4). These repeats are absent in FimV. However, the cytoplasmic region of *Pp*FimV is also highly enriched in acidic aa residues (25%, with 61 aspartic acid residues and 64 glutamic acid residues). The predicted pI of the cytoplasmic region is low (3.75), similar to that of the corresponding *Sp*HubP region (3.51).

**Fig 2:**
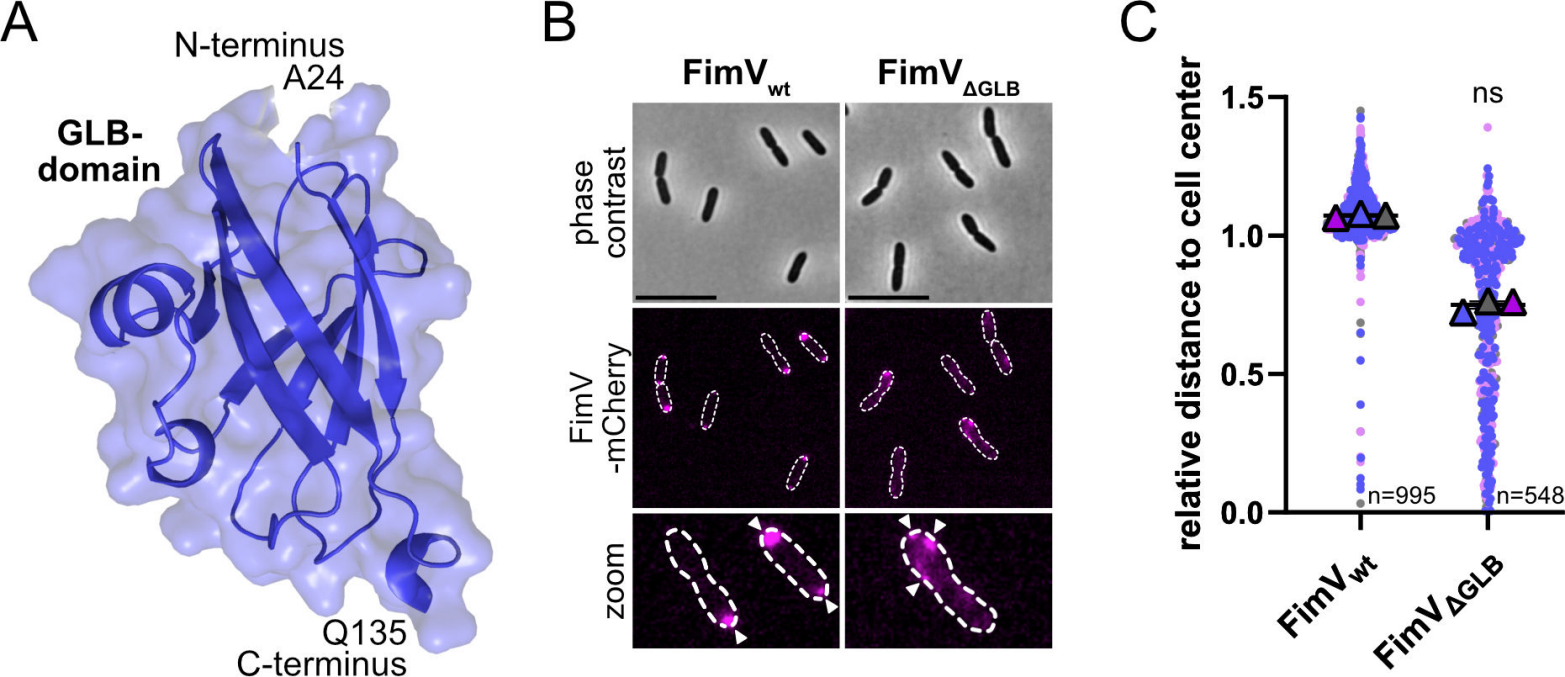
The periplasmic immunoglobulin-like domain is required for polar FimV positioning and function. (**A**) Alphafold prediction of immunoglobulin-like domain of FimV (Alphafold Database ID: AF-Q88LE1-F1-v6). N- and C-terminal amino acids are indicated. (**B**) Microscopic pictures of wild-type FimV (FimV_wt_) and deletion of immunoglobulin-like domain (FimV_ΔGLB_) carrying a translational fusion of FimV-mCherry (magenta). Arrows indicate fluorescent FimV cluster. Scale bar equals 5 µm. (**C**) Quantification of fluorescent FimV cluster. The distance (in micrometers) from a fluorescent FimV cluster to the cell center was measured and normalized to cell length. Values close to 1 indicate polar positioning, while values close to 0 indicate a cell center positioning. Data were generated using MicrobeJ (Fiji). The mean of each replicate (*N* = 3), and the corresponding mean of the means, together with the standard error of the mean, are shown. The total number of cells (*n*) for each strain is indicated. For statistical analysis, a Mann-Whitney test was performed (ns: non-significant, *P* = 0.1).

In addition to the aforementioned domains (SP, LysM-like, TM, and FimV), *Vc*HubP and *Sp*HubP possess predominantly unstructured regions interspersed with short alpha-helical sections, as predicted by AlphaFold3. By contrast, *Pp*FimV (and *Pa*FimV) harbor a second TPR domain within the cytoplasmic region (aa 487–556), which is absent from HubP. Furthermore, structure predictions by AlphaFold3 (27, 28) indicate the presence of a potential globulin-like additional domain (GLB) in the periplasmic region of FimV, located directly downstream of the predicted signal peptide (aa 24–135). This predicted domain is absent in *Vibrio* and *Shewanella* HubP (Fig. 1A and 2A, and Fig. S1). For peptidoglycan-binding analysis of the LysM domain, we used KT2442, a spontaneous rifampicin-resistant mutant of KT2440 (29). All further characterizations were carried out in a KT2440 strain that allowed fluorescent labeling of the flagellar filaments (FliC^S267C^). Previous studies showed that the substitution does not affect the swimming performance of the strain (30). Within this study, these strains are referred to as by their strain name (KT2442 or KT2440) and are considered to represent the wild type (WT).

### The periplasmic LysM domain of FimV interacts with peptidoglycan

FimV and HubP orthologs possess a LysM domain within their periplasmic region. LysM motifs are described to bind N-acetylglucosamine residues in peptidoglycan (PG), the main structural component of the bacterial cell wall (31) (Fig. S2A). Thus, we examined the ability of *Pp*FimV to interact with the PG layer by performing co-precipitation assays with PG sacculi isolated from *P. putida* cells using *P. putida* KT2442. Western blot analysis showed that purified *Pp*FimV-GFP co-precipitated *in vitro* with insoluble sacculi (Fig. 1B). Conversely, a *Pp*FimV-GFP version bearing a complete in-frame deletion of the predicted LysM-like domain (*Pp*FimVΔLysM-GFP) remained unbound and was recovered in the soluble fraction (Fig. 1B). These results suggest that FimV interacts with PG, and the LysM-like domain is essential to this interaction. Compared to the characteristic polar location of *Pp*FimV-GFP in *P. putida* cells, confocal microscopy showed that *Pp*FimVΔLysM-GFP is distributed in speckles along the cell contour (Fig. S2B), indicating that the mutant protein retains its ability to localize at the cell membrane, but the LysM-like domain is essential to polar anchoring. As GFP is not functional in the periplasm when transported via the cytoplasmic membrane via *sec*-dependent transport (32), the occurrence of fluorescent signals suggests that the mutant protein maintains its normal topology in the membrane. A co-immunoprecipitation assay using PG sacculi from *E. coli* (Fig. 1B, lower panel) revealed an interaction between *Pp*FimV-GFP and the sacculus, indicating that *Pp*FimV binding is not specific to *P. putida* PG.

Next, we aimed to delimit the minimal PG fragment recognized by FimV. To this end, co-precipitation assays were performed by incubating *Pp*FimV-GFP immobilized on an affinity matrix with a mixture of PG fragments released from sacculi digestion with different hydrolases. *V. cholerae*’s ShyA D,D-endopeptidase is described to cleave peptide bond crosslinks in PG (33). Accordingly, UPLC-MS/MS analysis showed that digestion of *V. cholerae* sacculus with *Vc*ShyA releases non-crosslinked polymeric PG strands of varying lengths (Fig. 1C). We found that *Pp*FimV-GFP was able to bind these PG strands, the shortest detected containing two N-acetylglucosamine/N-acetylmuramic acid disaccharide units (Fig. 1C). Although digestion of *P. putida* sacculi with *Vc*ShyA yielded a limited amount of soluble fragments (Fig. S2C), we detected a similar fraction of PG strands co-precipitated with *Pp*FimV-GFP (Fig. 1C). ShyA-digestion of *E. coli* sacculi released a very limited amount of soluble PG strands, and we did not detect any fraction co-precipitated with *Pp*FimV-GFP (Fig. S2D). Finally, we repeated the experiment using a mixture of *P. putida, V. cholerae, or E. coli* sacculus fragments obtained by digestion with muramidase, a lytic enzyme that cleaves the β-(1, 4)-glycosidic bond between N-acetylglucosamine and N-acetylmuramic acid, releasing non-crosslinked monomers, and dimeric, trimeric, or tetrameric crosslinked muropeptides (Fig. 1D; Fig. S2E and F) (34). In this case, we did not detect any species of *P. putida* muropeptides co-precipitated with *Pp*FimV-GFP, supporting the notion that the LysM-like domain of *Pp*FimV binds PG strands containing a minimum of two disaccharide units. In addition, we conclude that the PG binding by the *Pp*FimV LysM domain is not specific to *P. putida*, as it also occurs to PG of different gammaproteobacteria (*E. coli* and *V. cholerae*; Fig. 1B and C).

So far, there is no evidence that HubP or FimV is involved in global remodeling of the respective species’ cell walls. However, given the *Pp*FimV interaction with PG, we examined the PG composition in *P. putida* cells lacking FimV by muropeptide profiling after muramidase digestion as above. Differences were neither detected in the muropeptide profile of Δ*fimV* cells compared to the wild-type strain nor in PG amount, crosslinking abundance, or strand length (Fig. 1E; Fig. S2G), suggesting that FimV is not involved in global remodeling of the *P. putida* cell wall.

### A periplasmic immunoglobulin-like domain is required for polar FimV positioning

According to Alphafold structure predictions, the periplasmic region of *Pseudomonas* FimV harbors a section upstream of the LysM domain, which exhibits an immunoglobulin-like fold (Fig. 2A and Fig. S1). To investigate the potential function of this putative globulin domain (GLB), we created a mutant in which the native *fimV* gene was replaced with a *fimV::mCherry* hybrid containing an in-frame deletion of the domain-encoding gene segment (FimV_ΔGLB_-mCherry). This and all further studies were carried out in the reference strain *P. putida* KT2440. The protein fusion was stably produced (Fig. S3). Fluorescence microscopy revealed that the FimV variant had lost its defined localization pattern at the cell pole and division plane, instead exhibiting a speckled appearance (see Fig. 2B and C). Small clusters were often distributed along the cell envelope in proximity to one cell pole; however, FimV clusters also occurred away from the cell pole, close to the middle of the cell. Therefore, we concluded that the immunoglobulin-like domain within the periplasmic region of FimV, in addition to or together with the LysM-like domain, has a role in the proper localization of the protein.

### A role for FimV in flagella-mediated motility

Previous studies demonstrated that loss of *Pp*FimV results in a pronounced decrease in flagella-mediated swimming through soft agar (23). This loss of function, which similarly occurs in mutants lacking HubP (3, 4), may be caused by differences in flagella production, placement, and function, as well as chemotaxis defects and/or decreased cell growth. Previous studies have shown that lophotrichous flagellation is surprisingly unaffected in *ΔfimV* mutants of *P. putida* (23) (Fig. S4). Therefore, FimV plays only a minor role in establishing the lophotrichous flagellation pattern of *P. putida*.

#### Growth and morphology

To determine whether the decrease in swimming through soft agar is due to the effect of cell growth upon loss of FimV, wild-type and *ΔfimV* mutant cells were cultivated aerobically in planktonic cultures in either complex LB medium or mineral medium containing different carbon sources, such as amino acids, glucose, or fructose. No significant difference in growth occurred between the WT and the *fimV* mutant in all media (Fig. S5).

**Fig 3:**
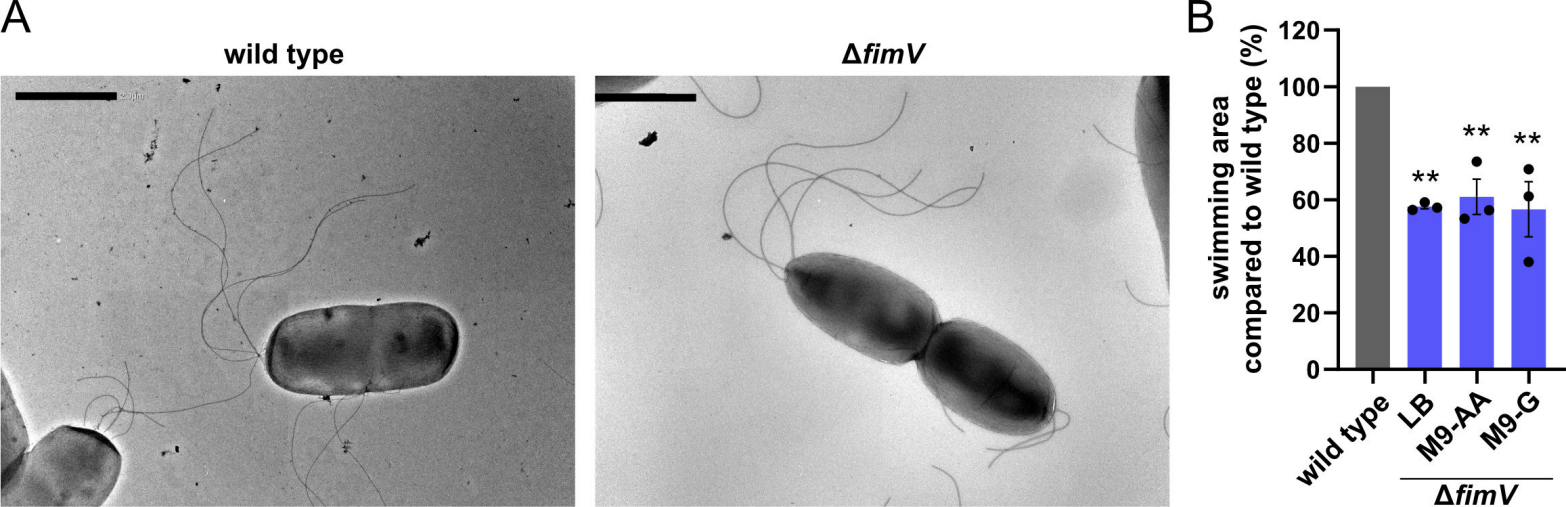
Deletion of *fimV* does not influence the flagellation pattern. (**A**) Transmission electron photograph showing cell morphologies of WT and *fimV* deletion mutant. Scale bar represents 2 µm. (**B**) Swimming behavior of *fimV* deletion compared to WT (set to 100%) in LB and M9 medium, supplemented with different carbon sources (AA: amino acids L-arginine and L-glutamine, G: glucose). Strains that were compared with each other were always spotted on the same plate. Data from three independent experiments with error bars are shown. For statistical analysis, one-way ANOVA was performed (**P<0.01, compared to WT).

Restricted movement through structured environments may be caused by irregular cell shapes. Therefore, we examined the general cell morphology of *P. putida* WT and Δ*fimV* mutant cells using transmission electron microscopy (TEM). No phenotypes concerning cell size or morphology were observed (Fig. 3A; Fig. S4C, in line with the observation that loss of *Pp*FimV does not apparently affect the peptidoglycan cross-linking (see Fig. 1E and Fig. S2G).

In *Vibrio vulnificus*, HubP recruits a positive regulator of flagellar assembly depending on the carbon source, thereby mediating motility in response to environmental nutrient conditions (15). To determine whether a similar mechanism may be active in *P. putida*, we assessed the ability of wild-type and *fimV* mutant cells to swim through soft agar in the presence of various carbon sources (the same ones used for growth experiments except fructose). We found that the decrease in the soft-agar swimming ability of *ΔfimV* mutants compared to that of the wild type (to about 60%) was independent of the medium condition (Fig. 3B, blue bars), suggesting that FimV does not mediate the regulation of flagellation in dependence of the carbon source in *P. putida*.

#### Localization of the chemotaxis cluster

**Fig 4:**
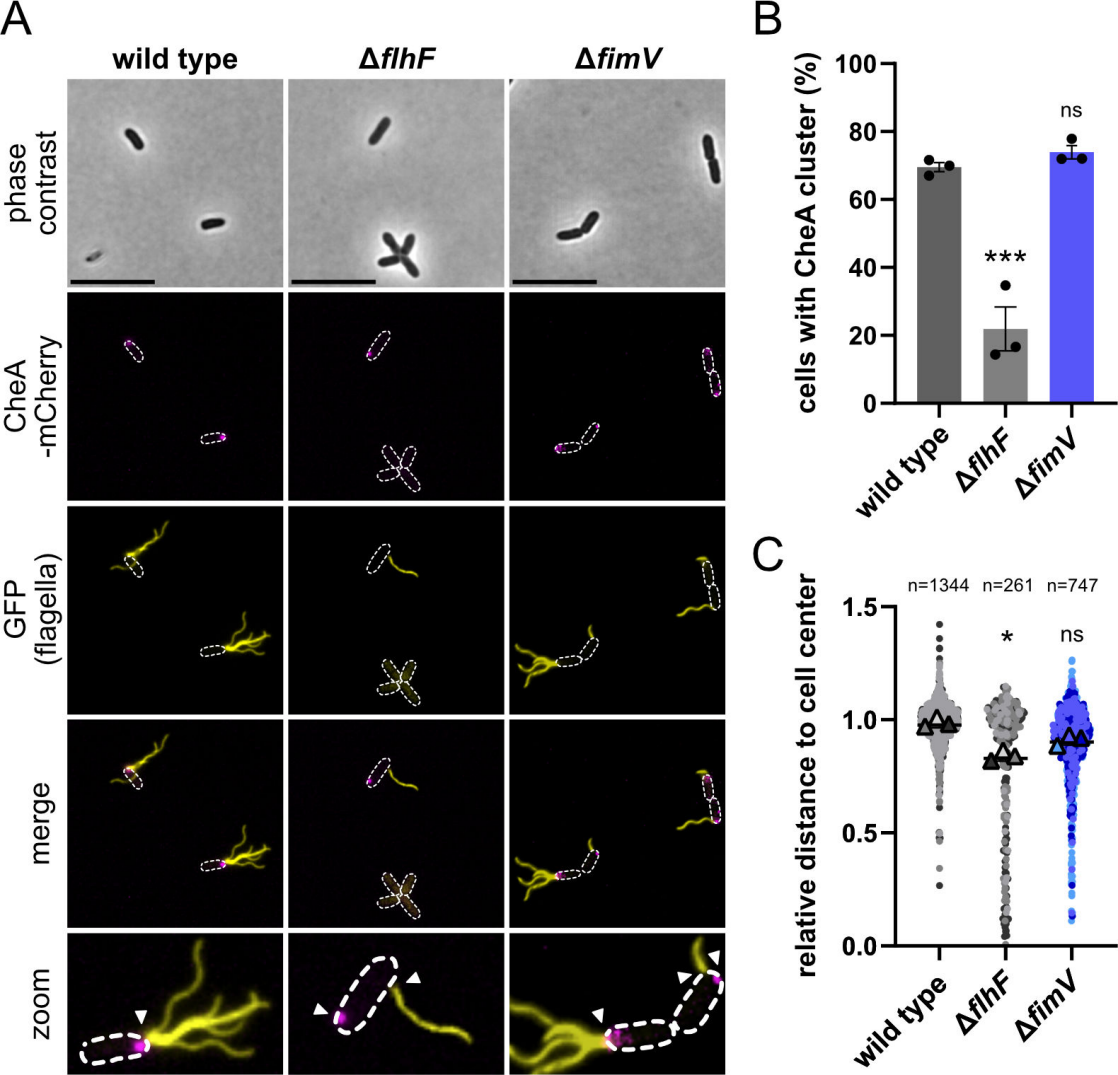
FimV has a minor effect on the positioning of the chemotaxis machinery. (**A**) Microscopic pictures of WT, *flhF,* and *fimV* deletion, carrying a translational fusion of CheA-mCherry (magenta), and staining of flagellar filaments (yellow) using Alexa 488 maleimide dye. Arrows indicate the position of the fluorescent CheA cluster or flagellar filaments. Scale bar equals 5 µm. (**B**) Percentage of cells with fluorescent CheA cluster. Data from three independent experiments with error bars are shown. For statistical analysis, one-way ANOVA was performed (***P<0.001, ns: non-significant (*P* = 0.4609), compared to WT). (**C**) Quantification of fluorescent CheA cluster. The distance (in µm) from a fluorescent CheA cluster to the cell center was measured and normalized to cell length. Values close to 1 indicate polar positioning, while values close to 0 indicate a cell center positioning. Data were generated using MicrobeJ (Fiji). The mean of each replicate (*N* = 3) and the corresponding mean of the means together with the standard error of the mean are shown. Total number of cells (*n*) for each strain is indicated. For statistical analysis, the Kruskal-Wallis test was performed (*P<0.05, ns: non-significant (*P*=0.3594), comparison to WT).

In *Shewanella* and *Vibrio*, HubP recruits the chemotaxis machinery to the flagellated cell pole, while the GTPase FlhF mediates positioning of the flagella in concert with the membrane-anchoring protein FipA (3, 4, 26). Previous studies have indicated that both the chemotaxis system and the flagellar machinery in *P. aeruginosa* are polarly positioned by the GTPase FlhF (35, 36). To test if this similarly applies to *P. putida*, we determined the localization of the chemotaxis system in a mutant lacking *flhF*. To this end, we fluorescently labeled CheA, the core component of the chemotaxis megacomplex (37, 38) by replacing *cheA* with a *cheA::mcherry* hybrid gene in the wild-type, *ΔflhF*, and *ΔfimV* mutant backgrounds. The fluorescent CheA-mCherry variant was stably produced (Fig. S3), still partly functional, as it supported swimming in soft agar to about 65% of that of WT CheA (Fig. S6) and therefore was suitable for fluorescent localization studies. We found that in *ΔflhF* mutants, less CheA protein is present and more aflagellate cells occur (Fig. 4A and B), which was expected as FlhF is required for the full expression of flagella and chemotaxis systems (23, 39). As reported for *P. aeruginosa* (35, 36), in *P. putida ΔflhF* cells that were still producing flagella and/or CheA clusters, these frequently occurred away from the cell pole. In addition, the co-localization of the CheA cluster and the flagella was lost (Fig. 4A). In *fimV* mutants, the flagellation pattern remained unchanged as had been established before (see above). CheA-mCherry generally localized to the flagellated cell pole in *fimV* mutants as in wild-type cells (Fig. 4C). However, we observed that the CheA localization was less distinct and occurred more often close to, but not directly at, or around the cell pole and was even split into several smaller subfoci (Fig. 4C). These results suggest that the polarity of the chemotaxis multiprotein complex partly depends on FlhF. FimV may be necessary for the accurate placement, assembly, and function of the chemotaxis machinery (Pulido-Sánchez et al. [40]).

### Loss of FimV does not affect chromosome segregation

**Fig 5:**
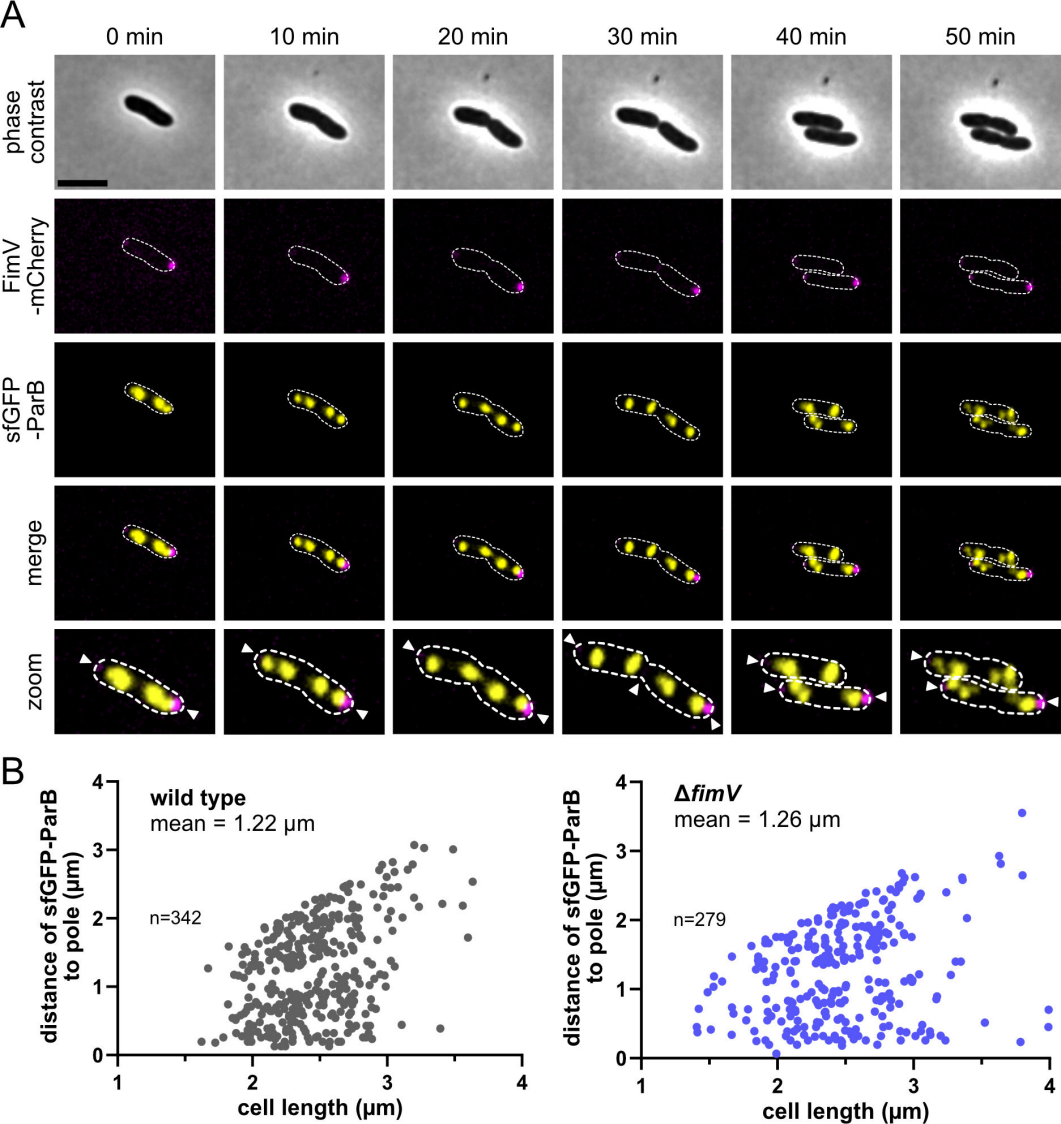
Loss of FimV does not affect chromosome segregation. (**A**) Fluorescent timelapse microscopy of cells carrying a translational fusion of FimV-mCherry (magenta) and sfGFP-ParB (yellow). Arrows indicate fluorescent FimV cluster. Scale bar equals 2 µm. (**B**) Distance (in µm) to fluorescent ParB cluster within the cells in the presence (left) or absence (right) of *fimV*. Measurements were generated using BacStalk software. Number of cells (*N*) together with the mean for each data analysis is indicated.

In addition to orchestrating normal swimming motility and chemotaxis, *Sp*HubP and *Vc*HubP have been shown to localize the chromosome’s origin of replication (*ori*) to the flagellated cell pole. To determine whether this also applies to *Pp*FimV, we localized the *ori* region of the chromosome in *P. putida* in the presence or absence of FimV. To visualize the *ori* region, we fluorescently labeled the ParB centromere-binding protein (PP_0001), which binds to the *parS* site on the chromosome close to the *ori*, by replacing the native *parB* gene with a *parB::sfgfp* hybrid gene. The ParB-sfGFP fusion protein was stably produced (Fig. S3) and formed distinct fluorescent foci within the cell (Fig. 5A). To enable the localization of the *ori* relative to FimV’s position *in vivo*, we used a background strain in which a chromosomal *fimV::mCherry* hybrid gene replaced the native *fimV* gene. We observed stable production of C-terminally mCherry-fused FimV, which enables flagella-mediated swimming in soft agar to nearly WT levels (Fig. S3 and S6). We then used time-lapse fluorescence microscopy to follow the position of the ParB-sfGFP foci over the cell cycle in the presence and absence of FimV (Fig. 5A). As expected, the position of the foci changed dynamically according to the progressing cell cycle and chromosome segregation. We observed that at the final position, ParB is not localized to the pole completely but remains at a position with a distance from the cell pole, which corresponds to approximately 10%–20% of the cell length (Fig. 5A and B). This distance to the pole was not significantly different between WT or *ΔfimV* mutant cells. Similarly, the temporal dynamics of ParB localization did not significantly differ between the strain backgrounds (Fig. 5B). Based on this, we concluded that FimV is not involved in chromosome segregation and polar localization in *P. putida*.

### FimV is not required for *P. putida* pilus-mediated surface motility

In *P. aeruginosa*, FimV is necessary for the polar targeting of the type IV pilus system. Accordingly, *fimV* mutants of this species are unable to perform pili-mediated surface movement, also known as twitching (15, 35). We investigated whether this role in surface motility is conserved in *P. putida*. Orthologous to the main building blocks of the *P. aeruginosa* type IV pilus machinery were readily identified in *P. putida,* with the exception of a clear homolog to the pilus extension ATPase PilB (41–45) (see Table S1). It is so far unclear if and how the *P. putida* T4P system functions, and to the best of our knowledge, twitching motility comparable to that of *P. aeruginosa* has not yet been demonstrated in this species.

First, we aimed to visualize the pilus filaments directly using TEM or fluorescent labeling from planktonic and agar surface-grown cultures, but no pilus-like structures were detected (data not shown). Additionally, we attempted to determine the presence and positioning of pilus complexes by fluorescently tagging the building blocks PilO, PilQ, or PilT. None of these protein fusions was detected by western blotting or by *in vivo* fluorescent microscopy (data not shown).

Therefore, we attempted to determine potential differences in pilus activity of *P. putida* or its *ΔfimV* mutant via pilus-mediated surface movement. To this end, we performed a twitching assay according to a protocol used for *P. aeruginosa*, which relies on the ability of the cells to move between agar and the bottom surface of a petri dish ([46]; see Materials and Methods section for details). To exclude a role of the flagella for motility, we used a strain bearing a deletion of *fliC*, which encodes the filament’s building block flagellin. In this background strain, we deleted *fimV* (*ΔfliC ΔfimV*). As a negative control, we created a strain lacking the gene encoding the major pilin, *pilA* (*ΔfliC ΔpilA*). For all strains, expansion of the colony between agar and bottom surfaces was observed (Fig. S7A), which had a similar appearance as reported for *P. aeruginosa* (19, 47). Notably, this expansion occurred indistinguishably in all strains tested, including that lacking *ΔfimV*. Thus, the observed expansion zone was formed independently of flagella and type IV pili and allowed no conclusions on a potential role of FimV in pilus activity.

**Fig 6:**
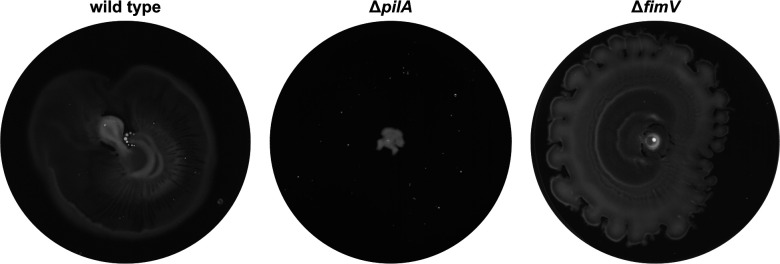
FimV is not required for pilus-mediated surface motility. Photographs of pilus-mediated surface motility in WT, *fimV,* and *pilA* deletion mutant on 0.5% PG-agar plates are shown. Cultures were incubated 2 days at 25°C after inoculation and re-inoculated on fresh PG-agar, followed by further incubation for 24 h. Representative pictures from at least three independent experiments are shown.

A previous study identified a pilus-dependent movement of *P. putida* across an agar surface resembling swarming motility that occurred independently of flagella (43). Using the same media (0.5% [wt/vol] Difco-agar, 0.5% Proteose-peptone No. 3, 0.2% [wt/vol] glucose), we successfully established the conditions under which this behavior occurred (see Materials and Methods section for details). We observed that the surface spreading ability of the *ΔfimV* mutant was highly similar to that of the WT but was absent in a mutant lacking *pilA* (Fig. 6). Thus, any type IV pilus activity required for moving across surfaces was unaffected in the absence of *fimV*. It should be mentioned that TEM indicated the presence of a pilus-like structure in surface-spreading of the *ΔfimV* mutant (Fig. S7B). However, this occurred in rare cases (less than 1 in 1,000 cells) and was not observed in WT spreading cells. Based on our findings, it cannot be excluded that FimV mediates the polar placement and/or activity of type IV pili in *P. putida*. However, pilus-dependent surface spreading pilus assembly and function appear unaffected by FimV.

### *Sp*HubP and its domains cannot functionally complement a *ΔfimV* mutant of *P. putida*

**Fig 7:**
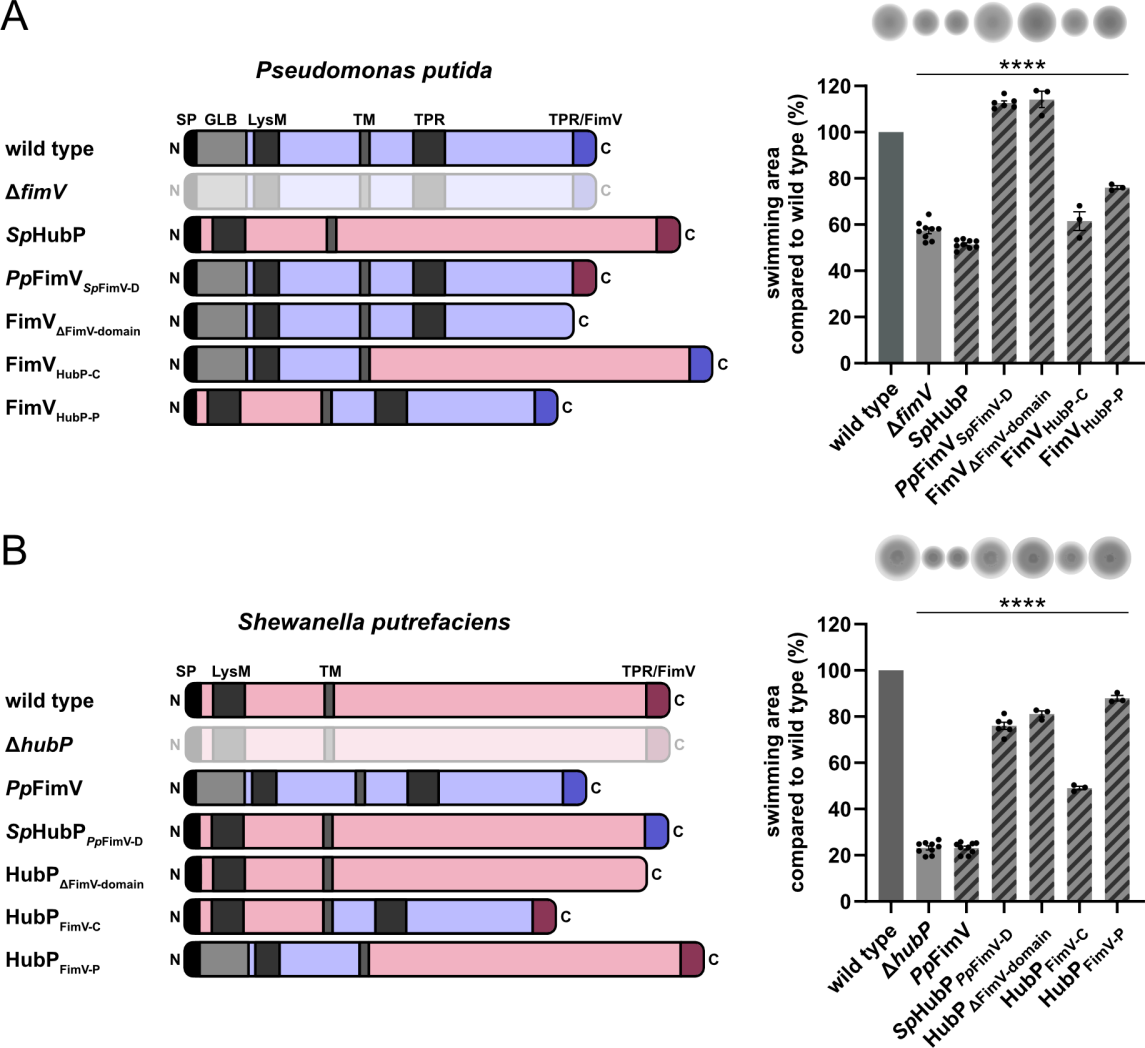
FimV cannot be functionally complemented by *Sp*HubP and its domains. Swimming behavior of *P. putida* (**A**) or *S. putrefaciens* (**B**) harboring different FimV variants with domains of the other species compared to the corresponding WT (set to 100%). Data from at least three independent experiments with error bars are shown. For statistical analysis, one-way ANOVA was performed (****P<0.0001, compared to corresponding WT). Schematic overviews represent protein structures with corresponding domains of *Pp*FimV and *Sp*HubP variants. N- and C-terminal orientation are indicated. The following abbreviations for protein domains are used: signal peptide (SP), immunoglobulin-like domain (GLB), LysM-like domain (LysM), transmembrane region (TM), and tetratricopeptide repeat-like domain (TPR/FimV).

Finally, given the structural similarities and differences between FimV and HubP, we investigated whether *Sp*HubP can complement a *ΔfimV* mutant in *P. putida*, and *vice versa*. Previous studies on *Pp*FimV have demonstrated its role in the polarity and function of the flagellar system; this is, so far, the only identified phenotype shared by *S. putrefaciens ΔhubP* and *ΔfimV* mutants in *P. putida* ([4]; Fig. S6). Ectopic *fimV* expression from a plasmid partially complemented the mutant, and reintegration of *fimV* into its native position fully restored the wild-type phenotype (Fig. S6C). To determine whether the swimming phenotype caused by deleting *fimV* could be complemented by *S. putrefaciens hubP*, we integrated a *hubP::sfgfp* hybrid gene into the chromosome, replacing *fimV* completely. *Sp*HubP-sfGFP and *Pp*FimV-sfGFP were stably produced (Fig. S3) and localized to the cell poles (Fig. S8), albeit weakly (*Pp*FimV-sfGFP in *S. putrefaciens*) or in a subpopulation (about 20%) of cells (*Sp*HubP-sfGFP in *P. putida*). *Sp*HubP production could not rescue the *ΔfimV* soft-agar swimming phenotype, and, *vice versa*, *Pp*FimV-sfGFP could not complement a *ΔhubP* phenotype, when the gene was integrated into the *hubP* locus within the *S. putrefaciens* chromosome (Fig. 7).

Since the full-length proteins of *Pp*FimV and *Sp*HubP were unable to functionally complement their respective counterparts, we asked whether single sections or domains could be exchanged between HubP and FimV. We began by swapping the native FimV C-terminal domain with the FimV domain of *S. putrefaciens*. Interestingly, the resulting *Pp*FimV*_Sp_*_FimV-D_ chimeric protein (Fig. 7A) was fully functional in terms of soft-agar swimming and performed slightly better than WT cells. Conversely, a HubP version with the C-terminal *Pp*FimV domain (*Sp*HubP*_Pp_*_FimV-D_; Fig. 7B) reduced the distance covered by swimming through soft agar to about 80%. As a control, we determined the function of *Pp*FimV and *Sp*HubP mutants, where we chromosomally deleted the C-terminal FimV domain. The truncated versions of *Pp*FimV and *Sp*HubP (FimV_ΔFimV-domain_ and HubP_ΔFimV-domain_, respectively) behaved exactly like the versions with the swapped FimV domain. We concluded from this that the FimV domains cannot be functionally swapped and that the C-terminal domain plays a minor role in mediating swimming motility.

Next, we swapped the cytoplasmic acidic domains located between the transmembrane (TM) domain and the C-terminal FimV domain between *Pp*FimV and *Sp*HubP (FimV_HubP-C_ and HubP_FimV-C_; Fig. 7A and B). In *P. putida*, the chimeric protein produced a phenotype similar to that of a complete *fimV* loss. In *S. putrefaciens*, the HubP_FimV-C_ chimera partially complemented the loss of *hubP* concerning swimming in soft agar, albeit to a low extent of about 50% of that of WT cells.

In the third step, we conducted the same experiment using the periplasmic domains of FimV and HubP (FimV_HubP-P_ in *P. putida* and HubP_FimV-P_ in *S. putrefaciens*; Fig. 7A and B). Once again, the chimeric FimV protein barely promoted swimming in soft agar beyond the levels observed in the *P. putida ΔfimV* strain. In contrast, the HubP version increased the swimming of the *ΔhubP* mutant from approximately 20% to nearly 80% of the WT level.

From these experiments, we concluded that the FimV domain plays a minor role in swimming through soft agar. Additionally, the acidic cytoplasmic and periplasmic sections of *P. putida* function surprisingly well in *S. putrefaciens* HubP. However, this is not the case *vice versa*.

## DISCUSSION

FimV (e.g., in *Pseudomonas*) and HubP (e.g., in *Vibrio* and *Shewanella*) are well-known polar landmark proteins that organize the bacterial cell poles via diffusion capture mechanisms. These proteins share a similar composition regarding major protein domains. They have not yet been identified as essential for any species, but FimV and HubP are involved in numerous cellular processes, some of which likely remain unidentified. Using *P. putida* FimV and *S. putrefaciens* HubP as models, we aimed to further understand the role and functional conservation of these widely conserved polar landmark proteins. We found that the periplasmic section *Pp*FimV harbors a conserved LysM-like domain, which binds the cell wall, and we newly identified a periplasmic globulin-like domain in the periplasmic section of *Pseudomonas* FimV that is absent in HubP. We established that both domains are required for normal positioning of *Pp*FimV. *Pp*FimV mutants exhibit a decrease in the ability to swim through soft agar, and we show that this is not due to flagellation, growth, general morphology, or medium conditions. Similar to *Vibrio* and *Shewanella* HubP, FimV appears to affect the proper localization, assembly, and, by that, the function of the chemotaxis cluster, which is likely one reason for the observed deficit in moving through soft agar. In contrast, we could not observe a role of FimV in chromosome segregation or for piliation, while it is not clear if, how, and when the T4P system of *P. putida* is active.

A key feature of HubP orthologs is their role in flagella-mediated motility. In their absence, the corresponding mutants typically exhibit a distinct phenotype concerning flagella-mediated swimming (3, 4, 15, 26). Previous studies by Pulido-Sanchez et al. (23) established that this similarly applies to *P. putida*. For example, cells exhibit a greatly reduced ability to swim through soft agar, which we used as a proxy for HubP/FimV function in this study. Previous studies have established that HubP and FimV are involved in the spatiotemporal regulation of polar flagellar system location and number in their respective species. This is mediated by an intricate interaction network involving FimV/HubP and the flagellar regulators FlhF and FlhG (FleN in *Pseudomonas*), as well as the placement factor FipA. This interaction network is present in both *S. putrefaciens* and *P. putida* (4, 23, 26, 48). However, the number and localization of flagella are surprisingly little affected by the loss of HubP or FimV in both species, which may be because part of the function can be mediated solely by FipA ([23]; Fig. 4A).

Another feature shared by HubP and *Pp*FimV that we identified here is the correct localization of the chemotaxis machinery to the flagellated cell pole. In HubP mutants of *V. cholerae*, *V. parahaemolyticus,* and *S. putrefaciens*, the chemotaxis supercomplex is displaced from the pole (3, 4, 11). In *P. putida*, the chemotaxis cluster remains at the flagellated cell pole in the absence of *fimV*, but its localization is less distinct than in WT cells (Fig. 4A and C), indicating that the localization and/or integrity of the cluster is disturbed. Together with the observation that *P. putida fimV* mutants display an aberrant swimming pattern involving fewer directional switches (23), this suggests that normal chemotaxis is disturbed in *P. putida fimV* mutants, which, we propose, is one of the main reasons for their poor swimming performance in soft agar. In *V. cholerae* and *V. parahaemolyticus*, the chemotaxis complex assembles at the flagellated pole via the ParC and ParP proteins (9–11). Potential orthologs of these proteins are present in *Shewanella* and *Pseudomonas* (39), and their role in chemotaxis cluster assembly in *P. putida* is characterized further in prepublished work (Pulido-Sánchez et al.(40)). Interestingly, deleting the C-terminal FimV domain of HubP or FimV, which is a major hub for client proteins (3–5, 16), did not affect the cells’ ability to spread in soft agar. Therefore, the placement of the chemotaxis system is likely mediated by the periplasmic domain or the acidic cytoplasmic part of HubP and FimV, respectively. We found that swapping the cytoplasmic domains resulted in a non-functional FimV chimera, whereas the HubP chimera exhibited partial functionality in *Shewanella* (Fig. 7). The role of this domain and the mechanism underlying its specificity are the subject of current studies. It needs to be noted that for *P. aeruginosa*, swimming through soft agar is not affected in the absence of FimV (19). Thus, a general role for FimV homologs for navigating through soft agar and, thus, for organizing the assembly of the chemotaxis system cannot be generalized even among Pseudomonads.

It had previously been established that both HubP and *Pp*FimV exhibit highly similar localization dynamics throughout the cell cycle (3, 4, 20). However, our cross-complementation assays, in which we replaced *fimV* with *hubP* and *vice versa,* suggested that normal localization was perturbed (Fig. S8). A defining feature of FimV and HubP is the presence of a periplasmic domain containing a LysM domain (Fig. 1A). First identified in the peptidoglycan-degrading lysozyme of *Bacillus* phage ɸ29 (49), this domain has since been found in all domains of life, including eukaryotes such as plants and fungi (50–52). LysM domains bind to N-acetylglucosamine-containing carbohydrates, such as peptidoglycan. In bacteria, most LysM domains are found in peptidoglycan-hydrolyzing proteins (52). Consistent with this, the LysM domain of *P. aeruginosa* FimV has been shown to interact with peptidoglycan (47). We similarly observed that *Pp*FimV interacts with *P. putida* PG through its predicted LysM domain (Fig. 1). Our results further indicate that *Pp*FimV PG binding is not species-specific, as it can also associate with PG from *E. coli* and *V. cholerae* (Fig. 1). The LysM-harboring periplasmic domains of *V. cholerae* and *S. putrefaciens* are required for the distinct localization pattern of HubP (3, 4). It is therefore likely that the intricate localization dynamics of FimV and HubP to the cell pole and division plane involve peptidoglycan binding. FimV was previously found to accumulate at midcell in predivisional *P. putida* cells (20), showing ring-like bands reminiscent of the ones displayed by the septal ring (53). Septal PG is enriched in denuded glycan strands stripped of the peptide stems, due to the enzymatic activity of cell wall amidases that mediate separation of the daughter cells during cell division (54). Our *in vitro* analyses indicate that *Pp*FimV can generally interact with PG strands holding their peptide stems, as long as they contain a minimum of two N-acetylglucosamine/N-acetylmuramic acid disaccharide residues (Fig. S2). Therefore, we consider it unlikely that the LysM domain can drive polar localization of *Pp*FimV by discriminating between lateral and septal PG. We propose that *Pp*FimV is recruited to the new pole through an as-yet-unidentified mechanism, but interaction with the cell wall stabilizes its polar anchoring.

Structural predictions of this protein section revealed an additional domain in *Pseudomonas* FimV that is absent from HubP: a periplasmic immunoglobular (GLB) domain located upstream of the LysM domain (Fig. 1A and 2A and Fig. S1). A FimV variant lacking this domain exhibited aberrant localization and loss of function regarding swimming motility (Fig. 2B), suggesting that it plays an important role in the accurate placement of FimV. Accordingly, swapping the periplasmic domains between *Sp*HubP and *Pp*FimV resulted in a HubP chimera that was active in *S. putrefaciens*, but in a FimV chimera that appeared to be almost inactive in *P. putida* (Fig. 7). Thus, the absence of this domain in the HubP periplasmic domain may be a reason why the domain is not functional in *P. putida*. Ig-like domains have long been shown to mediate the binding of diverse ligands, ranging from small molecules to other proteins (55). Therefore, we hypothesize that FimV requires another cellular structure or protein for proper polar placement. Further studies are required to identify potential binding partners of the FimV GLB- domain and elucidate its role in FimV localization and the function of the HubP counterpart without an apparent homologous periplasmic domain. Interestingly, the atypical FimV homolog in *Acinetobacter baumannii* completely lacks a periplasmic domain and, accordingly, localizes to and functions at lateral positions (56). FimV/HubP homologs are also present in non-rod-shaped cells, such as TspA of *Neisseria meningitidis* (57). Whether this protein marks the site of the previous cell division remains to be seen.

While the role of HubP and *Pp*FimV homologs in polarity and function of flagellation and chemotaxis appears to be conserved, we did not identify further functions that were previously identified for HubP orthologs and are shared by *Pp*FimV. *Pp*FimV is not apparently involved in chromosome segregation. Accordingly, we found that in *P. putida*, the chromosomal *parS* region is not completely localizing to the cell pole as in *V. cholerae* or in *S. putrefaciens* (3, 4) (Fig. 5), and this has similarly been shown previously for *P. aeruginosa* (58). Similarly, we found no indication that *Pp*FimV mediates flagellation in response to the carbon source as in *V. vulnificus* (15) (Fig. 3; Fig. S5). While we used a limited set of carbon sources in this study, the regulatory components of *V. vulnificus* and *P. putida* involved in catabolite repression are fundamentally different (15, 59). Thus, we conclude that similar modes of regulation are rather unlikely. HubP from *V. cholerae* has also been reported to polarly localize the cell wall remodeling enzyme DacB, a bifunctional DD-endopeptidase/carboxypeptidase (14). In the same species, deletion of the LysM domain causes *Vc*HubP to localize along the cell contour without altering cell morphology (3), similar to our observations in *P. putida* cells. While we did not detect any changes in morphology or PG composition in the Δ*fimV* mutant (Fig. 3A; Fig. S2G), we do not discard that *Pp*FimV can mediate polar recruitment of unidentified client proteins involved in local PG remodeling in *P. putida*.

A major role previously demonstrated for FimV/HubP homologs is to mediate T4P activity. FimV was first identified in *P. aeruginosa* to be required for normal T4P-mediated twitching (7), and subsequent studies provided evidence that FimV is the central hub to govern *P. aeruginosa* T4P polar localization and activity (16, 18, 19, 47, 60). Additionally, the FimV/HubP homologs of *Legionella pneumophila*, *Neisseria meningitidis*, and *Acinetobacter baumannii* are implicated in pilus assembly or activity (56, 57, 61). However, we could not verify a similar role for FimV in *P. putida*. Generally, the function and potential roles of the *P. putida* Pil T4P system are mostly unknown. Proteome analysis on *P. putida* demonstrated the presence of the PilA subunit in 12h-old biofilms, while PilA was absent in planktonic cultures (62). However, the role of the Pil system in surface attachment or biofilm formation is currently unknown. *P. putida* has, so far, not been demonstrated to exhibit twitching motility, and in this study, we were also unable to do so. In contrast, the species’ T4P system has been implicated to be required for another form of surface motility, a flagella-independent swarming-like movement across an agar surface (43). The role of the pili in this type of movement is currently unknown. The same study identified pilus-like appendages on moving cells by TEM analysis, which were not polarly localized, but distributed around the cell body (43). Such appendages were rarely seen in our study and occurred solely on *ΔfimV* mutants. In addition, *fimV* mutants similarly displayed the pilus-dependent swarming-like surface movement (Fig. 6), suggesting that, whatever the function of the pili may be in this means of surface spreading, FimV has no role in it.

In summary, we found that the polar landmark proteins *Pp*FimV and *Sp*HubP only share some key functions, for example, in flagella-mediated motility and chemotaxis. It is even unlikely that the FimV landmarks of *P. aeruginosa* and *P. putida* have a similar role in pilus assembly or flagella/chemotaxis functions, indicating that these polar markers have evolved a functional diversification even in more closely related species. HubP/FimV homologs are present in numerous species among the proteobacteria, and it will be interesting to trace the evolution and the mechanism underlying the functional diversification of this group of polar landmark proteins.

## MATERIAL AND METHODS

### Strains, media, and growth conditions

For the cloning experiments, *E. coli* cells were grown in LB medium at 37°C, while *P. putida* cells were grown at 30°C throughout the day or at room temperature overnight. Medium containing 50 μg/mL kanamycin, 300 μM 2.6-diaminopimelic acid, and 12% (wt/vol) sucrose was used for conjugation or plasmid-based *fimV* expression. Medium containing 10 μg/mL gentamycin was used for selection of Tn7 transposon insertion.

### Generation of strains

The bacterial strains, plasmids, and oligonucleotides used in this study are listed in Tables S2 to S4. *E*. *coli* DH5αλ*pir* was used for cloning the respective derivatives of plasmid pNPTS138-R6K for sequential crossover as previously described using Gibson Assembly (63, 64). *E. coli* WM3064 was used for the introduction of DNA into *P. putida*. Site-specific integration of miniTn*7* derivatives in *P. putida* was performed as previously described (65, 66).

### Electroporation

For ectopic *fimV* expression, the broad-host-range vector pBBR1-MCS2 was used as backbone. A cumate-inducible system (*cymR*/P*_cym_*) from *Pseudomonas putida* F1 was introduced into the construct. For electroporation, 2 mL of stationary-phase culture was washed twice with 300 mM sorbitol and 200 ng of plasmid DNA was used for electroporation (2.5 kV voltage, 25 μF capacitance, and 200 Ω resistance). Cells were recovered for 2 h at 30°C prior to induction (61). *fimV* expression was induced with 2 mM cumate.

### Growth experiments

To investigate the growth behavior, strains were diluted in LB medium or M9 minimal medium with different carbon sources (0.4% glucose, fructose, or 5 mM each of L-arginine and L-glutamine) to an optical density of 0.02 and incubated at 30°C for 48 h in Epoch2 microplate reader (BioTek) under constant shaking. Measurements were taken every 30 min at 600 nm.

### Motility assays

For swimming motility of *P. putida* on soft agar, 2 µL of an exponentially growing culture were spotted onto 0.25% LB-agar plates (select agar, Invitrogen). If necessary, antibiotics were added to the agar. Plates were incubated at 30°C for 18 h. Swimming areas were measured using ImageJ. Strains that were compared with each other were always spotted on a common plate to ensure a direct comparison. For pilus-mediated surface motility assays across agar surfaces (43), 2 µL of a stationary phase culture were spotted onto 0.5% PG-agar plates (0.5% Difco-agar, 0.5% Proteose-peptone No. 3, 0.2% Glucose [wt/vol]) and incubated for 48 h at 25°C. Afterward, cells from the edge of the motile fraction were reinoculated onto fresh PG-agar plates and incubated for 24 h at 25°C. For putative twitching motility, stationary phase cultures were adjusted to an OD_600_ of 1.5. The agar (1% LB-agar made with select agar, Invitrogen) was pierced with a sterile Pasteur pipet, and 1 µL of the adjusted cultures was spotted onto the bottom of the plates. Plates were incubated for 24 h at 30°C. After incubation, the agar was removed, and twitching zones were stained using 0.5% crystal violet (wt/vol) for 30 min. Plates were carefully washed with water to remove crystal violet stain and dried at room temperature. Twitching zones were measured using ImageJ. Strains that were compared with each other were always spotted on a common plate to ensure a direct comparison. An Epson V700 photo scanner was used for documentation of all plates.

### Flagella staining

Flagella fluorescent labeling was carried out as described before (30). A serine-to-cysteine substitution at position 267 was inserted into the gene encoding the flagellin gene *fliC* to stain the flagellar structures (FliC^S267C^). This strain was used throughout the study except the peptidoglycan analysis. Cells were harvested from exponentially growing cultures and always handled with cut pipette tips to avoid shear forces on extracellular structures. Cells were gently centrifuged at 1,200 × *g* for 5 min, and the pellet was resuspended in 1× PBS. For staining, the fluorescent dye Alexa Fluor 488-C5-maleimide (ThermoFisher Scientific) was added and incubated for about 20 min in the dark. The cells were then washed twice with 1× PBS to remove unbound dye. Cells were analyzed by fluorescence microscopy as described below.

### Fluorescence microscopy

Fluorescence microscopy was used to detect intracellular fusion proteins or extracellular structures. For this purpose, the cells were spotted onto 1× PBS agarose pads (select agar, Invitrogen). When appropriate, membranes were stained by incubating cells in PBS buffer containing 5 µg/mL FM 4-64 (ThermoFisher Scientific) for 5 min in the dark. Fluorescent microscopy was carried out as previously described (4), using a microscope set-up based on a Leica DMI 6000 B inverse microscope, equipped with a pco.edge sCMOS camera (PCO), a SPECTRA light engine (lumencor), an HCPL APO 63×/1.4–0.6 objective (Leica) using a custom filter set (T495lpxr, ET525/50m; Chroma Technology), and the VisiView software application (Visitron Systems, Puchheim, Germany). Alternatively, cells were imaged using an Axio Observer7 confocal microscope (Zeiss), equipped with a CSU-W1 spinning disk module (Yokogawa), an α Plan-Apochromat 100×/1.46 Oil DIC M27 inverted objective (Zeiss) and a Prime 95B CMOS camera (Teledyne Photometrics) controlled by SlideBook 6 software, using 488 and 514 nm excitation laser lines combined with 525/50 and 617/73 nm filters, respectively.

### Transmission electron microscopy

For negative staining and transmission electron microscopy (TEM), cells were collected either from exponentially growing liquid cultures or PG-agar plates. For liquid cultures, glow-discharged Formvar/carbon-coated copper grids (300 mesh) were placed on a 10 µL drop of bacterial suspension for 15 min. Alternatively, grids were placed briefly on cells grown on PG-agar plates. Grids were rinsed three times with distilled water and stained with 2% uranyl acetate for 30 s. Grids were then examined using a JEM-1400 TEM (JEOL), operated at 100 kV.

### Western blot analysis

Western blot experiments were performed to check for expression and stability of the fusion proteins. For this purpose, cells were harvested from exponentially growing cultures and adjusted to the same optical density (OD_600_ of 10). To separate the protein lysate by SDS-PAGE, 10 µL of the samples were loaded onto the gel. The proteins were then transferred to PVDF membranes by western blotting and visualized with primary antibodies against mCherry or GFP. A luminescent signal was generated using CDP-Star chemiluminescent substrate (Roche, Switzerland) or SuperSignal West Pico PLUS Chemiluminescent Substrate (Thermo Fisher) and secondary antibodies coupled to alkaline phosphatase. The signal was detected with the Fusion-SL chemiluminescence imager (Peqlab, Erlangen, Germany) or using an ImageQuant LAS 4000 imager (GE Healthcare).

### Protein expression and purification

Overnight LB cultures were diluted to an OD_600_ of 0.01 and grown to an OD_600_ of 0.3. Protein expression was induced by adding 2 mM sodium salicylate when appropriate. When reaching OD_600_ of 1, cells were collected by centrifugation (5,000 × *g*, 4°C, 15 min) and resuspended in cold buffer A (20 mM Tris-HCl pH 8.0, 500 mM NaCl, 1% Nonidet P-40). Cells were disrupted by sonication, and the soluble fraction was rotating-incubated overnight at 4°C with ChromoTek GFP-Trap Agarose beads previously equilibrated with buffer A. Beads were washed 12 times with buffer A without Nonidet P-40, and protein elution was performed by vortex resuspension of the agarose beads in 200 mM glycine pH 2.5 for 1 min. Supernatant was transferred to a new tube and neutralized with 200 mM Tris-HCl pH 8.0.

### Peptidoglycan preparations

Peptidoglycan sacculi from *E. coli* and *P. putida* cells were isolated as previously described (67). Samples of *V. cholerae* sacculi were kindly supplied by Prof. Felipe Cava’s lab.

To obtain non-crosslinked peptidoglycan strands, 20 mg of saccule preparations were digested overnight at 37°C with 5 µM *Vc*ShyA in ShyA buffer (20 mM Tris-maleate pH 6.8, 30 mM NaCl, 10 mM MgCl2, 1 mM DTT, 0.1% Triton X-100). Purified *Vc*ShyA was a gift from Prof. Felipe Cava’s lab. Reactions were stopped by boiling, and samples were centrifuged and transferred to new tubes. Muropeptides were obtained by sacculus digestion with muramidase (80 mg L^-1^) at 37°C overnight as previously described (67).

### Co-precipitation assays

To analyze protein interaction with total peptidoglycan, co-precipitation assays with purified saccule were performed essentially as described (47), incubating 5 µg of purified protein with 2 mg of sacculi in PBS buffer in ice for 2 h. Proteins in pellets and soluble fractions were subsequently analyzed by western blot.

To analyze interaction with soluble peptidoglycan fragments, 25 µL of GFP-Trap agarose beads (ChromoTek) bearing immobilized *Pp*FimV-GFP or GFP (1 mg mL^-1^ of protein) were incubated in ice for 2 h with 25 µL of non-reduced muramidase/ShyA-derived peptidoglycan fragments. Beads were washed three times with PBS buffer, and pulled-down fragments were released by boiling. Samples were centrifuged, and supernatants were further subjected to reduction and analysis by UPLC-MS/MS.

### Peptidoglycan analysis

Mixtures obtained after ShyA or muramidase treatment were subjected to reduction with sodium borohydride as previously described (68). Chromatographic separation of reduced peptidoglycan samples was performed as previously described (67), using an Acquity H-Class UPLC system (Waters) equipped with an Acquity UPLC BEH C18 column (Waters), monitoring A_204_. Separation was achieved with a linear gradient from phase A (0.1% formic acid) to phase B (0.1% formic acid, 40% acetonitrile) over 18 min at a flow rate of 250 µL min^-1^. Peaks were identified using a Xevo G2-XS QTOF mass spectrometer and by comparison of retention times. Data acquisition, processing, and integration of the peaks were carried out using the UNIFI software (Waters). Total peptidoglycan abundance was estimated as the area below the UPLC chromatogram. Relative amount of each identified muropeptide was calculated as the peak area divided by total area. Percentage of crosslinkage was calculated as % of dimers + (% of trimers × 2). Average chain length was calculated as 100 divided by the percentage of anhydromuropeptides.

### Data analysis

Microscopic and swimming images were analyzed using ImageJ (v1.54p). Growth experiments were performed using Gen5 3.10. Graph creation and statistics were done using Prism 10.4.2 (GraphPad software) or BacStalk 1.8stable (64).

### Bioinformatic tools

To predict the domain organization, cellular localization, and properties of FimV and HubP, we used the following tools: SignalP 6.0 (https://services.healthtech.dtu.dk/services/SignalP-6.0/; (69) to predict the presence of N-terminal signal peptides and DeepTMHMM (https://services.healthtech.dtu.dk/services/DeepTMHMM-1.0/; (70)) to predict the presence and position of potential transmembrane regions and the positioning of the domains relative to the cytoplasmic membrane. The pI was computed by the Expasy pI/Mw tool (https://web.expasy.org/compute_pi/; (71)). General information about the proteins was gathered from the UniProt database (https://www.uniprot.org/), also using the domain prediction databases linked therein.

## SUPPLEMENTAL MATERIAL

Supplemental tables and figures jb.00497-25-s0001.pdf Tables S1 to S4 and Figures S1 to S8.

ASM does not own the copyrights to Supplemental Material that may be linked to, or accessed through, an article. The authors have granted ASM a non-exclusive, world-wide license to publish the Supplemental Material files. Please contact the corresponding author directly for reuse.
