# Supplementary material for: Functional characterization of the polar organizer protein FimV in Pseudomonas putida
Source: J Bacteriol. 2026 Jan 12;208(2):e00497-25. doi: 10.1128/jb.00497-25 (PMC12918733; doi:10.1128/jb.00497-25)
Supplement: Supplemental tables and figures — Tables S1 to S4 and Figures S1 to S8. [file jb.00497-25-s0001.pdf]

Functional characterization of the polar organizer protein FimV in *Pseudomonas putida*

Lisa Marie Schmidt[#], Marta Pulido-Sánchez[#], Anke Treuner-Lange, Lukas Zehner, Aroa López-Sánchez, Felipe Cava, Fernando Govantes[*], and Kai M Thormann[*]

Supplementary Information

contains: **Supplementary Tables S1 – S4**

Supplementary Figures S1 – S8

Supplementary Tables

Supplementary Table 1. Gene homologs of *P. putida* and *P. aeruginosa*. *pil* genes with their corresponding gene numbers in *P. putida* and *P. aeruginosa*, respectively.

Gene name	*P. putida*	*P. aeruginosa*
pilA	PP_0634	PA4525
pilB	no documented homolog	PA4526
pilC	PP_0633	PA4527
pilD	PP_0632	PA4528
pilE	PP_0611	PA4556
pilF	PP_0851	PA3805
pilG	PP_4992	PA0408
pilH	PP_4991	PA0409
pilI	PP_4990	PA0410
pilJ	PP_4989	PA0411
pilK	no documented homolog	PA0412
pilM	PP_5083	PA5044
pilN	PP_5082	PA5043
pilO	PP_3480	PA5042
pilP	PP_5081	PA5041
pilQ	PP_5080	PA5040
pilT	PP_5093	PA0395
pilU	no documented homolog	PA0396

Supplementary Table S2. Bacterial strains used in this study

Escherichia coli

Strain	Genotype	Reference
DH5α	*sup E44, ΔlacU169 (ΦlacZΔM15), recA1, endA1, hsdR17, thi-1, gyrA96, relA1*	(1)
DH5αλ*pir*	*sup E44, ΔlacU169 (ΦlacZΔM15), recA1, endA1, hsdR17, thi-1, gyrA96, relA1, λpir phage lysogen*	(2)
WM3064	*thrB1004 pro thi rpsL hsdS lacZ ΔM15 RP4-1360 Δ(araBAD) 567ΔdapA 1341: [erm pir(wt)]*	W. Metcalf, University of Illinois, Urbana-Champaign

Pseudomonas putida

Strain	Genotype	Reference
Wild type	wild type strain of *P. putida* KT2440	(3)
KT2442	spontaneous rifampicin resistant mutant of *P. putida* KT2440, used for PG analysis.	(4)
Δ*fliC*	deletion of the gene *fliC* (PP_4378).	(5)
Δ*pilA*	deletion of the gene *pilA* (PP_0634).	This study
Δ*fliC* Δ*pilA*	deletion of the gene *fliC* (PP_4378) and deletion of the gene *pilA* (PP_0634).	This study
Δ*fliC* Δ*fimV*	deletion of the gene *fliC* (PP_4378) and deletion of the gene *fimV* (PP_1993).	This study
$FliC^{S267C}$	markerless in-frame substitution of Ser267 to Cys in the flagellin gene *fliC* (PP_4378).	(5)
Δ*fimV*	markerless in-frame substitution of Ser267 to Cys in the flagellin gene *fliC* (PP_4378), deletion of the gene *fimV* (PP_1993).	This study
Δ*fimV* (KT2442)	deletion of the gene *fimV* (PP_1993) in KT2442, used for PG analysis.	(6)
$FimV_{\Delta GLB}$	markerless in-frame substitution of Ser267 to Cys in the flagellin gene *fliC* (PP_4378), deletion of immunoglobulin-like domain in *fimV* (PP_1993, ΔL30-Q135).	This study
p*fimV*	markerless in-frame substitution of Ser267 to Cys in the flagellin gene *fliC* (PP_4378), deletion of the gene *fimV* (PP_1993), harboring cumate-inducible pBBR1 plasmid with *fimV* gene	This study
Δ*fimV::fimV*	markerless in-frame substitution of Ser267 to Cys in the flagellin gene *fliC* (PP_4378), complementation of the deleted *fimV* gene (PP_1993) into the native locus.	This study
*Sp*HubP	markerless in-frame substitution of Ser267 to Cys in the flagellin gene *fliC* (PP_4378), expression of *S. putrefaciens hubP* (Sputcn32_2442) in *P. putida fimV* deletion mutant.	This study
*Sp*HubP-3xGGS-sfGFP	markerless in-frame substitution of Ser267 to Cys in the flagellin gene *fliC* (PP_4378), expression of *S. putrefaciens hubP* (Sputcn32_2442) fused to	This study

	sfGFP with 3x Gly, Gly and Ser in *P. putida fimV* deletion mutant.	
*Pp*Fim$V_{SpFimV\text{-}D}$	markerless in-frame substitution of Ser267 to Cys in the flagellin gene *fliC* (PP_4378), expression of *P. putida fimV* (PP_1993) with *S. putrefaciens* HubP FimV-domain (Sputcn32_2442, A1034-S1097).	This study
Fim$V_{HubP\text{-}C}$	markerless in-frame substitution of Ser267 to Cys in the flagellin gene *fliC* (PP_4378), expression of *P. putida fimV* (PP_1993) with *S. putrefaciens* HubP C-terminal part (Sputcn32_2442, L336-G1033).	This study
Fim$V_{HubP\text{-}P}$	markerless in-frame substitution of Ser267 to Cys in the flagellin gene *fliC* (PP_4378), expression of *P. putida fimV* (PP_1993) with *S. putrefaciens* HubP periplasmic-terminal part (Sputcn32_2442, P34-N313).	This study
Fim$V_{\Delta FimV\text{-}domain}$	markerless in-frame substitution of Ser267 to Cys in the flagellin gene *fliC* (PP_4378), deletion of C-terminal FimV domain within *fimV* (PP_1993, ΔG861-V911).	This study
FimV-3xGGS-mCherry	markerless in-frame substitution of Ser267 to Cys in the flagellin gene *fliC* (PP_4378), expression of *fimV* (PP_1993) fused to mCherry with 3x Gly, Gly and Ser.	This study
Fim$V_{\Delta GLB}$-3xGGS-mCherry	markerless in-frame substitution of Ser267 to Cys in the flagellin gene *fliC* (PP_4378), expression of *fimV* (PP_1993) fused to mCherry with 3x Gly, Gly and Ser without immunoglobulin-like domain (ΔL30-Q135).	This study
KT2442 *attTn7::*P*sal-fimV-gfp*	expression of *fimV* (PP_1993) fused to GFPmut3 from the salicylate inducible P*sal* promoter into the *att7 locus.*	(6)
KT2442 *attTn7::*P*sal-fimV*ΔLysM-*gfp*	expression of *fimV* (PP_1993) bearing a deletion of LysM domain fused to GFPmut3 from the salicylate inducible P*sal* promoter into the *att7 locus.*	This study
CheA-GS-mCherry	markerless in-frame substitution of Ser267 to Cys in the flagellin gene *fliC* (PP_4378), expression of *cheA* (PP_4338) fused to mCherry with Gly and Ser.	This study
CheA-GS-mCherry Δ*fimV*	markerless in-frame substitution of Ser267 to Cys in the flagellin gene *fliC* (PP_4378), expression of *cheA* (PP_4338) fused to mCherry with Gly and Ser, deletion of the gene *fimV* (PP_1993).	This study
CheA-GS-mCherry Δ*flhF*	markerless in-frame substitution of Ser267 to Cys in the flagellin gene *fliC* (PP_4378), expression of *cheA* (PP_4338) fused to mCherry with Gly and Ser, deletion of the gene *flhF* (PP_4343).	This study
sfGFP-2xGGS-ParB	markerless in-frame substitution of Ser267 to Cys in the flagellin gene *fliC* (PP_4378), expression of	This study

	parB (PP_0001) fused to sfGFP with 2x Gly, Gly and Ser.	
sfGFP-2xGGS-ParB Δ*fimV*	markerless in-frame substitution of Ser267 to Cys in the flagellin gene *fliC* (PP_4378), expression of *parB* (PP_0001) fused to sfGFP with 2x Gly, Gly and Ser, deletion of the gene *fimV* (PP_1993).	This study
sfGFP-2xGGS-ParB FimV-3xGGS-mCherry	markerless in-frame substitution of Ser267 to Cys in the flagellin gene *fliC* (PP_4378), expression of *parB* (PP_0001) fused to sfGFP with 2x Gly, Gly and Ser, expression of *fimV* (PP_1993) fused to mCherry with 3x Gly, Gly and Ser.	This study

Shewanella putrefaciens

**Strain**	**Genotype**	**Reference**
Wild type	wild type strain of *S. putrefaciens* CN-32	(7)
Δ*hubP*	deletion of the gene *hubP* (Sputcn32_2442).	(8)
*Pp*FimV	expression of *P. putida fimV* (PP_1993) in *S. putrefaciens hubP* deletion mutant.	This study
*Pp*FimV-3xGGS-sfGFP	expression of *P. putida fimV* (PP_1993) fused to sfGFP with 3x Gly, Gly and Ser in *S. putrefaciens hubP* deletion mutant.	This study
*Sp*HubP$_{\mathit{Pp}\text{FimV-D}}$	expression of *S. putrefaciens hubP* (Sputcn32_2442) with *P. putida* FimV-domain (PP_1993, P848-V911).	This study
HubP$_{\text{FimV-C}}$	expression of *S. putrefaciens hubP* (Sputcn32_2442) with *P. putida fimV* C-terminal part (PP_1993, R410-D852).	This study
HubP$_{\text{FimV-P}}$	expression of *S. putrefaciens hubP* (Sputcn32_2442) with *P. putida fimV* periplasmic part (PP_1993, L25-N386).	This study
HubP$_{\Delta\text{FimV-domain}}$	deletion of C-terminal FimV domain within *hubP* (Sputcn32_2442, ΔE1047-S1097).	This study

Supplementary Table S3. Plasmids used in this study

General

Plasmid	Description	Reference
pNPTS138-R6KT	mobRP4+ ori-R6K *sacB;* β-galactosidase fragment alpha; suicide vector for *in-frame* deletions or integrations in *P. putida*; Kan^r	(9)
pBBR1-MCS2-cym	Integration of *P. putida* F1 cym-system (cymR/Pcym) into pBBR1-MCS2 vector backbone, Kan^r	This study
pUC18Sfi-miniTn*7*BB-Gm	pUC18SfiI-based delivery plasmid for the synthetic minitransposon miniTn*7*BB-Gm. Ap^r Gm^r	(10)
pMRB189	pUC18Sfi-miniTn*7*BB-Gm derivative for expression of C-terminal *gfp*-mut3 translational fusions under the salicylate inducible P*sal* promoter; Ap^r Gm^r	(6)
pTNS2	ori-R6K; helper plasmid for expression of *Tn7* transposase; Ap^r	(9)

Plasmids for *Pseudomonas putida*

Plasmid	Description	Reference
pBBR1-MCS2-cym-pFimV	Ectopic expression of *fimV* upon induction of cumate-inducible cym-system from *P. putida* F1, Kan^r	This study

Plasmids for protein overexpression in *Escherichia coli* DH5α

Plasmid	Description	Reference
pBK-miniTn*7-gfp2*	pUC19-based delivery plasmid for miniTn*7*-ΩGm transposon containing a P*A1/04/03-gfp*mut3 transcriptional fusion. Ap^r Gm^r	(11)
pMRB236	Ectopic expression of FimV-GFP upon induction of the P*sal* promoter in pMRB189. Ap^r Gm^r	(6)
pMRB401	Ectopic expression of FimVΔLysM-GFP upon induction of the P*sal* promoter in pMRB189. Ap^r Gm^r	This study

Plasmids for *Escherichia coli* DH5αλpir

Plasmids	Description	Reference
pNPTS138-R6KT Δ*fimV*	deletion of the *fimV* gene (PP_1993); Kan^r	This study
pNPTS138-R6KT $FimV_{ΔGLB}$	Deletion of immunoglobulin-like domain within *fimV* (PP_1993, ΔL30-Q135); Kan^r	This study
pNPTS138-R6KT Δ*flhF*	deletion of the *fimV* gene (PP_4343); Kan^r	This study

pNPTS138-R6KT Δ*fimV::fimV*	in frame complementation of the *fimV* gene (PP_1993); Kan^r	This study
pNPTS138-R6KT Δ*fliC*	deletion of the *fliC* gene (PP_4378); Kan^r	(5)
pNPTS138-R6KT Δ*pilA*	deletion of the *pilA* gene (PP_0634); Kan^r	This study
pNPTS138-R6KT FliC^{S267C}	in frame complementation of Ser267 to Cys in the flagellin gene *fliC* (PP_4378); Kan^r	(5)
pNPTS138-R6KT FimV-3xGGS-mCherry	in frame complementation of *fimV* (PP_1993) with FimV-3xGGS-mCherry; Kan^r	This study
pNPTS138-R6KT CheA-GS-mCherry	in frame complementation of *cheA* (PP_4338) with CheA-GS-mCherry; Kan^r	This study
pNPTS138-R6KT sfGFP-2xGGS-ParB	in frame complementation of *parB* (PP_0001) with sfGFP-2xGGS-ParB; Kan^r	This study
pNPTS138-R6KT *Sp*HubP	in frame complementation of the *fimV* gene (PP_1993) with *hubP* (Sputcn32_2442); Kan^r	This study
pNPTS138-R6KT *Sp*HubP-3xGGS-sfGFP	in frame complementation of the *fimV* gene (PP_1993) with *hubP*-3xGGS-*sfgfp* (Sputcn32_2442); Kan^r	This study
pNPTS138-R6KT *Pp*FimV$_{\textit{Sp}\text{FimV-D}}$	in frame complementation of the *fimV* gene (PP_1993) with the C-terminal FimV domain of *hubP* (Sputcn32_2442, A1034-S1097); Kan^r	This study
pNPTS138-R6KT FimV$_{\text{HubP-C}}$	in frame complementation of the *fimV* gene (PP_1993) with the C-terminal part of *hubP* (Sputcn32_2442, L336-G1033); Kan^r	This study
pNPTS138-R6KT FimV$_{\text{HubP-P}}$	in frame complementation of the *fimV* gene (PP_1993) with the periplasmic part of *hubP* (Sputcn32_2442, P34-N313); Kan^r	This study
pNPTS138-R6KT FimV$_{\Delta\text{FimV-domain}}$	deletion of the C-terminal FimV domain within *fimV* gene (PP_1993, ΔG861-V911); Kan^r	This study
pNPTS138-R6KT *Pp*FimV	in frame complementation of the *hubP* gene (Sputcn32_2442) with *fimV* (PP_1993); Kan^r	This study
pNPTS138-R6KT *Pp*FimV-3xGGS-sfGFP	in frame complementation of the *hubP* gene (Sputcn32_2442) with *fimV*-3xGGS-*sfgfp* (PP_1993); Kan^r	This study
pNPTS138-R6KT *Sp*HubP$_{\textit{Pp}\text{FimV-D}}$	in frame complementation of the *hubP* gene (Sputcn32_2442) with the C-terminal FimV domain of *fimV* (PP_1993, P848-V911); Kan^r	This study
pNPTS138-R6KT HubP$_{\text{FimV-C}}$	in frame complementation of the *hubP* gene (Sputcn32_2442) with the C-terminal part of *fimV* (PP_1993, R410-D852); Kan^r	This study
pNPTS138-R6KT HubP$_{\text{FimV-P}}$	in frame complementation of the *hubP* gene (Sputcn32_2442) with the periplasmic part of *fimV* (PP_1993, L25-N386); Kan^r	This study
pNPTS138-R6KT HubP$_{\Delta\text{FimV-domain}}$	deletion of the C-terminal FimV domain within *hubP* gene (Sputcn32_2442, ΔE1047-S1097); Kan^r	This study

Supplementary Table S4. Oligonucleotides used in this study

Name	Sequence
**Sequencing/Check primer**	
M13 fwd	TGTAAAACGACGGCCAGTCC
M13 rev	CACACAGGAAACAGCTATGACC
check CheA-mCh fwd	GCCACCAGTTTGTAGGGTTTGCG
check CheA-mCh rev	CCGAGATTTCCGATGTGTCCGG
check fimV fwd-1	GCACGGTCATCGACCTGTCC
check fimV fwd-2	GAAGCTCAGCCGGAAGAAC
check fimV fwd-3	GACCAGCAGCAGATCCAGAG
check fimV rev-1	CGGGTCGTTGTAGATGACATAGC
check fimV rev-2	ATGTCGATATAGGCACGGGC
check fimV rev-3	CGTGCCAACTGGTTGTTCTT
check flhF fwd	CGCGTAGGCGTCGGTAATCG
check flhF rev	GCCTAAACTTGCAGAAGAGCTGG
check hubP fwd-1	GCTAAAGAACCTAACACCACGA
check hubP fwd-2	GGGAATGGCAAGCATGAC
check hubP rev-1	TCCTGTTGGTGTTCATTACCA
check hubP rev-2	GTAAAGAACCCCACGTGCTG
check parB fwd	TGTTCAAGGCGTGCGATATC
check parB rev	GTATCAATCTCGCCGCCTC
check fliC fwd	ACGCTTTGAAGAGCAACACC
check fliC rev	AGTGATCTGCTGTTGCTTGG
check S267C fwd	ATCAAAGGTCTGAGCGTTGC
check S267C rev	CTCGGCTCTACCAACATTGC
seq fimV-1	ATAGCGTGCTGCTCGACCAG
seq fimV-2	CGGCTGTGGTTGCCGCTTC
seq fimV-3	CGACTTGCCATCGGACTTCGAC
seq fimV-4	TGCCACTTTTTCCGCAGC
seq fimV-5	ATCAGACAGGTCCACGGC
seq fimV-6	GGAAAGCCTGGACACCAG
seq fimV-7	TGCACGATGAGCCCAAAG
seq fimV-8	GGTGCCAGCAGCTACACC
seq SpHubP$_{PpFimV-D}$-1	GATGCGAGTGTGAGTGTCTATC
seq SpHubP$_{PpFimV-D}$-2	GATGATGATCTCGATTTAAGCA
seq FimV$_{HubP-C}$	GAAATGTTTATCGATACAGGCAG
seq sfGFP	CTGGAGTTGTCCCAATTCTT
tn7-glmS	AATCTGGCCAAGTCGGTGAC
tn7-R109	CAGCATAACTGGACTGATTTCAG
**Construction Δ*fliC***	
EcoRV fliC fwd	GAATTCGTGGATCCAGATGCAATACGGTGTCCTCCAGTG
OL fliC-KO rev	CGAGCAGCTTAGCCATGACGAATTCCTCGTTG
OL fliC-KO fwd	CGTCATGGCTAAGCTGCTCGGCTAATTCGATTC
EcoRV fliC rev	CAAGCTTCTCTGCAGGATAGCCGATACCACTGACTGTTG
**Construction Δ*pilA***	
EcoRV pilA fwd	CAAGCTTCTCTGCAGGATTCGCAACCACTGCGG
OL pilA-KO rev	TGGTTTATTGCCCTGCCCGAACGTAGTTCCTTTTTGATTCGC
OL pilA-KO fwd	CAAAAAGGAACTACGTTCGGGCAGGGCAATAAACCA
EcoRV pilA rev	GAATTCGTGGATCCAGATCTTATGCCTGTCGGATCTACGTC
**Construction FliC^{S267C}**	
OL fliC-S267C rev	GCTGTCAATGCAAGCGGTAACACC

OL fliC-S267C fwd	TTACCGCTTGCATTGACAGCGCC
**Construction Δ*fimV* and Δ*fimV::fimV***	
EcoRV fimV fwd	GAATTCGTGGATCCAGATCCAGGTGGCATTCAACCTGC
OL fimV-KO rev	TCAGACCAGCCGAAGCATGACCTCTTCCCTTG
OL fimV-KO fwd	TCATGCTTCGGCTGGTCTGATGCAAGCAGG
EcoRV fimV rev	CAAGCTTCTCTGCAGGATCTTGAAGCGGGCATGAAAGTCG
**Construction FimV$_{\Delta GLB}$**	
EcoRV GLB fwd	CAAGCTTCTCTGCAGGATCTGGGCCTGCCTGAACTG
OL GLB-KO rev	TGCCGCCGGCTGTTCGCCCTCCCCCAGGCCCAG
OL GLB-KO fwd	GCGCTGGGCCTGGGGGAGGGCGAACAGCCGGC
EcoRV GLB rev	GAATTCGTGGATCCAGATTCTTCGTTGTCGCGACG
**Construction p*fimV***	
SmaI cym-FimV fwd	AATTCGAGCTCGGTACCCATGCTTCGAATTCGCAAACTG
SmaI cym-FimV rev	CGACTCTAGAGGATCCCCTCAGACCAGCCGGGAGA
**Construction Δ*flhF***	
EcoRV flhF fwd	GAATTCGTGGATCCAGATGAACGAAGCTGACCACAGAGTC
flhF-KO rev	GCAAGTTAAGGGTTGACCATGAAGCGTGTGC
flhF-KO fwd	ATGGTCAACCCTTAACTTGCATTATCCCCTACCTC
EcoRV flhF rev	CAAGCTTCTCTGCAGGATGCTGCAAGCCCTGTTGTCGG
**Construction FimV-3xGGS-mCherry**	
EcoRV OL FimV fwd	CAAGCTTCTCTGCAGGATATTTCGACCTCGACGTCAGC
OL FimV rev	CGAACCCCCGCTACCGCCGCTACCGCCGACCAGCCGGGAGAGC
FimV-mCh fwd	GGCGGTAGCGGCGGTAGCGGGGGTTCGGTTTCCAAAGGGGAAGAGGAC
FimV-mCh rev	CCTGACCTGCTTGCATCATTTGTATAACTCATCCATACCACCAGTC
OL FimV fwd	ATGGATGAGTTATACAAATGATGCAAGCAGGTCAGG
EcoRV OL FimV rev	GAATTCGTGGATCCAGATGCGGGCATGAAAGTCG
**Construction *Sp*HubP in KT2440**	
EcoRV OL FimV fwd	GCCAAGCTTCTCTGCAGGATGAAAGTCGGCCGGCATTG
OL FimV rev	GAGATTAGTTAATGCAAGCAGGTCAGGCC
OL HubP fwd	GACCTGCTTGCATTAACTAATCTCTTTTAGTAAACGTCCGG
OL HubP rev	AGGGAAGAGGTCATGAAATTTCGCACTTCGTATCTTGT
OL FimV fwd	GCGAAATTTCATGACCTCTTCCCTTGTATGAATCGT
EcoRV OL FimV rev	GCGAATTCGTGGATCCAGATGCTGGCCCAGGTCGG
**Construction *Sp*HubP-3xGGS-sfGFP in KT2440**	
EcoRV OL HubP fwd	CAAGCTTCTCTGCAGGATATCTGAGTGATGACAGTGTTCTTGC
EcoRV OL HubP rev	CGAACCCCCGCTACCGCCGCTACCGCCACTAATCTCTTTTAGTAAACGTCCGG C
OL FimV-sfGFP fwd	GGCGGTAGCGGCGGTAGCGGGGGTTCGATGAGCAAAGGAGAAGAACTTTT CAC
OL FimV-sfGFP rev	CCTGACCTGCTTGCATTAGGATCCTTTGTAGAGCTCATCCAT
EcoRV OL FimV fwd	GAGCTCTACAAAGGATCCTAATGCAAGCAGGTCAGGCC
EcoRV OL FimV rev	GAATTCGTGGATCCAGATAAAGTCGGCCGGCATTG
**Construction *Pp*FimV$_{SpHubP\text{-}D}$ in KT2440**	
EcoRV PPFimV fwd	CAAGCTTCTCTGCAGGATTTCAACCTGCTGGCCCAG
PPFimV-SpFimVdomain rev	GGCATCGCCAAGTAAGGCCTCTGGTTGCGCCACC
PPFimV-SpFimVdomain fwd	GCGGTGGCGCAACCAGAGGCCTTACTTGGCGATGCC

SpFimVdomain-PPFimV rev	AGGCCTGACCTGCTTGCATTAACTAATCTCTTTTAGTAAACGTCCGG
PPFimV OL fwd	CTAAAAGAGATTAGTTAATGCAAGCAGGTCAGGCC
PPFimV OL rev	GAATTCGTGGATCCAGATCTTGAAGCGGGCATGAAAGTCG

Construction FimV$_{HubP-C}$ in KT2440

EcoRV OL fimV fwd	CAAGCTTCTCTGCAGGATAGCTGGATGCCACTCGC
OL HubP-C rev	ATTACGTTTACGACGAAGCGCCAGCAGCCACAG
HubP-C fwd	CTGCTGTGGCTGCTGGCGCTTCGTCGTAAACGTAATAAAGAGCG
HubP-C rev	GAAGTCGAAGTCCAGGTCACCAACGTCACTCATCTCAATGT
OL Hub-PC fwd	GAGATGAGTGACGTTGGTGACCTGGACTTCGACTTCTTCTCC
EcoRV OL fimV rev	GAATTCGTGGATCCAGATAATCGGCTCGTTGGCG

Construction FimV$_{HubP-P}$ in KT2440

EcoRV OL fimV fwd	CAAGCTTCTCTGCAGGATGAACTGCGCGTACTGCTGG
OL HubP-P rev	TAAAGGTTTTACTTTAGGCGCATTCGCCATGCC
HubP-P fwd	TCTGGCATGGCGAATGCGCCTAAAGTAAAACCTTTAAAAATCATGGG
HubP-P rev	CACGCCCAGAAGCCACGGATTATTGATAATCTTCCGCCATAAGTCA
OL HubP-P fwd	CGGAAGATTATCAATAATCCGTGGCTTCTGGGC
EcoRV OL fimV rev	GAATTCGTGGATCCAGATGGCCGGCAACTGACG

Construction FimV$_{\Delta FimV\text{-}domain}$ in KT2440

EcoRV PpFimV-d fwd	CAAGCTTCTCTGCAGGATTCGCTGAGCCTGTGCTC
PpFimV-d-KO rev	CCTGACCTGCTTGCATCAGGAGAAGAAGTCGAAGTCCAGGT
PpFimV-d-KO fwd	GACTTCGACTTCTTCTCCTGATGCAAGCAGGTCAGG

Construction *Pp*FimV in CN-32

EcoRV OL HubP fwd	GCCAAGCTTCTCTGCAGGATCTTGGTGCATTTTGTCGGCA
OL HubP rev	CGGCTGGTCTGATCTCGATTAACCGAGTTTCAATCTAAC
OL FimV fwd	GGTTAATCGAGATCAGACCAGCCGGGAGA
OL FimV rev	AGGGAAGGATTGATGCTTCGAATTCGCAAACTGG
OL HubP fwd	AATTCGAAGCATCAATCCTTCCCTTTGAAGCGC
EcoRV OL HubP rev	GCGAATTCGTGGATCCAGATTAGTGAATGCGACAGCTGTACG

Construction *Pp*FimV-3xGGS-sfGFP in CN-32

OL FimV-sfGFP fwd	GGCGGTAGCGGCGGTAGCGGGGGTTCGATGAGCAAAGGAGAAGAACTTTT CAC
OL FimV-sfGFP rev	CTCGGTTAATCGAGATCAGGATCCTTTGTAGAGCTCATCCAT
OL FimV fwd	GAGCTCTACAAAGGATCCTGATCTCGATTAACCGAGTTTCAATC
OL FimV rev	GAATTCGTGGATCCAGATCCGTGATAATGGCTTACACCATG

Construction *Sp*HubP$_{PpFimV\text{-}D}$ in CN-32

EcoRV SpHubP fwd	CAAGCTTCTCTGCAGGATTGAATGCGACAGCTGTACG
SpHubP-PPFimVdomain rev	GTCGTCCAAAGGCTCAGGACCAACGTCACTCATCTCAATG
SpHubP-PPFimVdomain fwd	GAGATGAGTGACGTTGGTCCTGAGCCTTTGGACGAC
PPFimVdomain-SpHubP rev	AAACTCGGTTAATCGAGATCAGACCAGCCGGGAG
SpHubP OL fwd	CTCTCCCGGCTGGTCTGATCTCGATTAACCGAGTTTCAATCTAAC
SpHubP OL rev	GAATTCGTGGATCCAGATTTACCGTGATAATGGCTTACACC

Construction HubP$_{FimV\text{-}C}$ in CN-32

EcoRV OL HubP fwd	CAAGCTTCTCTGCAGGATTTGTTAAGGTTGAACCTAAAACTGAGC

OL fimVC rev	CTGGGCCTTGCGTTTGCGCATCATAAATACAAGTAATAAAATCAGTAGCGC
fimVC fwd	TTACTTGTATTTATGATGCGCAAACGCAAGGCC
fimVC rev	GGCATCGCCAAGTAAGGCGTCCAAAGGCTCAGGCTCTG
OL fimVC fwd	GAGCCTGAGCCTTTGGACGCCTTACTTGGCGATGCC
EcoRV OL HubP rev	GAATTCGTGGATCCAGATATTGCATTCGTATCAAAATGCACC
**Construction HubP$_{FimV\text{-}P}$ in CN-32**	
EcoRV OL hubP fwd	CAAGCTTCTCTGCAGGATTACTGCGTAATGCCGAAGGA
OL FimV-P rev	CAGCTCCCCCAGGCCCAGCGTATCTGCAGCAACAGCG
FimV-P fwd	GCTGTTGCTGCAGATACGCTGGGCCTGGGGGAG
FimV-P rev	TGCAATTAACATTGCAGGGTTGCCCAGGACCGAGTC
OL FimV-P fwd	GACTCGGTCCTGGGCAACCCTGCAATGTTAATTGCAGCC
EcoRV OL hubP rev	GAATTCGTGGATCCAGATGCCAAGTCCCGCCAG
**Construction HubP$_{\Delta FimV\text{-}domain}$ in CN-32**	
EcoRV SpFimV-d fwd	CAAGCTTCTCTGCAGGATTCACACCAACCCTCGATG
SpFimV-d-KO rev	CTCGGTTAATCGAGATTAATCATCGACATCAATTATAGCGG
SpFimV-d-KO fwd	ATAATTGATGTCGATGATTAATCTCGATTAACCGAGTTTCAATC
EcoRV SpFimV-d rev	GAATTCGTGGATCCAGATCCGTGATAATGGCTTACACCA
**Construction sfGFP-2xGGS-ParB**	
EcoRV OL ParB fwd	CAAGCTTCTCTGCAGGATTGCAGGTCAAGGAAAGCC
OL GFP rev	TTCTTCTCCTTTGCTCATACGGATTCCTTAAGTTGTTTGTGC
GFP ParB fwd	ACAACTTAAGGAATCCGTATGAGCAAAGGAGAAGAACTTTTCAC
GFP ParB rev	GCTACCGCCGCTACCGCCGGATCCTTTGTAGAGCTCATCCA
OL GFP fwd	GGCGGTAGCGGCGGTAGCATGGCCGTCAAGAAACGG
EcoRV OL ParB rev	GAATTCGTGGATCCAGATGCCACAGTCACCCGCG
**Construction CheA-GS-mCherry**	
EcoRV_OL_PP_4338_fwd	GAATTCGTGGATCCAGATCGTGGTGAAAACCAAGATCTCCC
OL_PP_4338_rev	TGGAAACCATGCTACCAATACGCCGCGCGGCGTAAC
PP_4338-mCh_fwd	GCGGCGTATTGGTAGCATGGTTTCCAAAGGGGAAGAGGAC
PP_4338-mCh_rev	ACCGAAATCATTTGTATAACTCATCCATACCACCAGTC
OL_PP_4338_fwd	GTTATACAAATGATTTCGGTGGCGCGCCCT
EcoRV_OL_PP_4338_rev	CAAGCTTCTCTGCAGGATCTGCGCCTGGCTGGTAAAGG
**Construction pMRB401**	
FimV-SpeI-RBS_fwd	ACGTACTAGTGAAAGAGGAGAAATACTAGATGCTTCGAATTCGCAAACTG
FimV-LysM-OL_rev	AGGCGACGGCCTTCACGCCATCCACCCTGGGTGTTACG
FimV-LysM-OL_fwd	GTAACACCCAGGGTGGATGGCGTGAAGGCCGTCG
FimV-BamHI_rev	ACGTGGATCCGACCAGCCGGGAGAGCAT

Abbreviations: fwd: forward; rev: reverse

Additional References Tables S2 & S3

1. Hanahan D. 1983. Studies on transformation of *Escherichia coli* with plasmids. J Mol Biol 166:557–580.
2. Miller VL, Mekalanos JJ. 1988. A novel suicide vector and its use in construction of insertion mutations: osmoregulation of outer membrane proteins and virulence determinants in *Vibrio cholerae* requires *toxR*. J Bacteriol 170:2575–2583.
3. Nelson KE, Weinel C, Paulsen IT, Dodson RJ, Hilbert H, Martins dos Santos V a. P, Fouts DE, Gill SR, Pop M, Holmes M, Brinkac L, Beanan M, DeBoy RT, Daugherty S, Kolonay J, Madupu R, Nelson W, White O, Peterson J, Khouri H, Hance I, Chris Lee P, Holtzapple E, Scanlan D, Tran K, Moazzez

A, Utterback T, Rizzo M, Lee K, Kosack D, Moestl D, Wedler H, Lauber J, Stjepandic D, Hoheisel J, Straetz M, Heim S, Kiewitz C, Eisen JA, Timmis KN, Düsterhöft A, Tümmler B, Fraser CM. 2002. Complete genome sequence and comparative analysis of the metabolically versatile *Pseudomonas putida* KT2440. Environ Microbiol 4:799–808.

4. Franklin FC, Bagdasarian M, Bagdasarian MM, Timmis KN. 1981. Molecular and functional analysis of the TOL plasmid pWWO from *Pseudomonas putida* and cloning of genes for the entire regulated aromatic ring meta cleavage pathway. Proc Natl Acad Sci USA 78:7458–7462.
5. Hintsche M, Waljor V, Großmann R, Kühn MJ, Thormann KM, Peruani F, Beta C. 2017. A polar bundle of flagella can drive bacterial swimming by pushing, pulling, or coiling around the cell body. Sci Rep 7:16771.
6. Pulido-Sánchez M, Leal-Morales A, López-Sánchez A, Cava F, Govantes F. 2025. Spatial, temporal and numerical regulation of polar flagella assembly in *Pseudomonas putida*. Microbiol Res 292:128033.
7. Fredrickson JK, Zachara JM, Kennedy DW, Dong H, Onstott TC, Hinman NW, Li S. 1998. Biogenic iron mineralization accompanying the dissimilatory reduction of hydrous ferric oxide by a groundwater bacterium. Geochim Cosmochim Acta 62:3239–3257.
8. Rossmann F, Brenzinger S, Knauer C, Dörrich AK, Bubendorfer S, Ruppert U, Bange G, Thormann KM. 2015. The role of FlhF and HubP as polar landmark proteins in *Shewanella putrefaciens* CN-32. Mol Microbiol 98:727–742.
9. Lassak J, Henche A-L, Binnenkade L, Thormann KM. 2010. ArcS, the cognate sensor kinase in an atypical Arc system of *Shewanella oneidensis* MR-1. Appl Environ Microbiol 76:3263–3274.
10. Jiménez-Fernández A, López-Sánchez A, Calero P, Govantes F. 2015. The c-di-GMP phosphodiesterase BifA regulates biofilm development in *Pseudomonas putida*. Environ Microbiol Rep 7:78–84.
11. Lambertsen L, Sternberg C, Molin S. 2004. Mini-Tn*7* transposons for site-specific tagging of bacteria with fluorescent proteins. Environ Microbiol 6:726–732.

Supplementary Figures

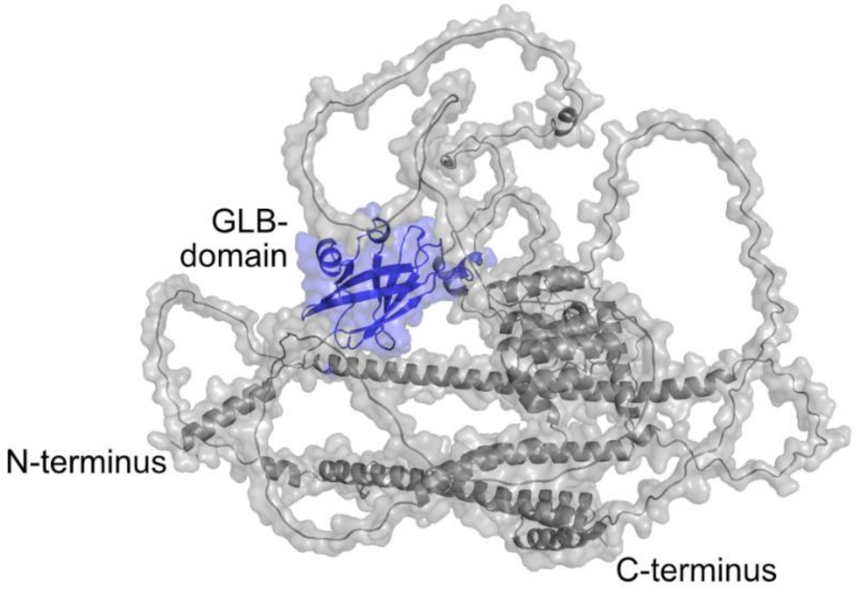

Supplementary Figure S1. FimV Alphafold prediction. Alphafold prediction showing cartoon with surface structure of *P. putida* KT2440 FimV (PP_1993, grey) with indicated immunoglobulin-like domain (GLB-domain, blue) and N- and C-terminus, respectively (Alphafold Database ID: AF-Q88LE1-F1-v6).

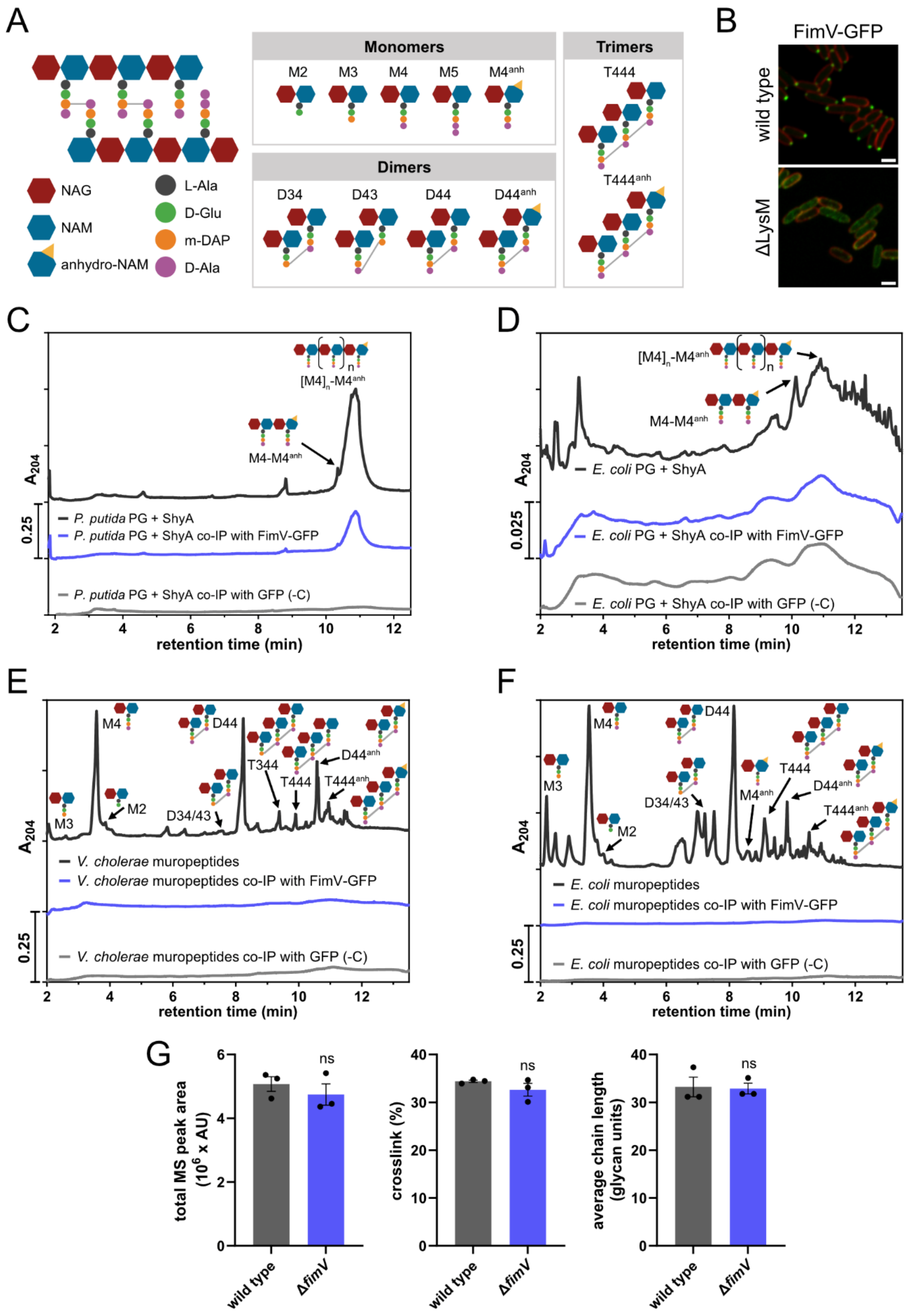

Supplementary Figure S2. FimV interacts with peptidoglycan cell wall. A Peptidoglycan composition. NAG: N-acetylglucosamine; NAM: N-acetylmuramic acid; m-DAP: meso-diaminopimelic acid. **B** Confocal microscopy images of WT *P. putida* cells bearing a P*sal-fimV-gfp* or P*sal-fimV*ΔLysM-*gfp* transposon in the *att7* locus (green). FM™ 4-64 was used as membrane stain (red). Images are shown as the maxima projections of seven Z-sections of the green channel, merged with the red channel

showing the cell contour at the focal plane. Scale bar equals 2 µm. **C** UPLC chromatograms showing soluble PG fragments released by *P. putida* sacculus digestion with *Vc*ShyA, and the fragments co-immunoprecipitated with FimV-GFP and GFP (-C). An arrow denotes *Vc*ShyA cleavage. **D** UPLC chromatograms showing soluble PG fragments released by *E. coli* DH5α sacculus digestion with *Vc*ShyA, and the fragments co-immunoprecipitated with FimV-GFP and GFP (-C). **E** UPLC chromatograms showing soluble PG fragments released by *V. cholerae* O1 sacculus digestion with muramidase, and the fragments co-immunoprecipitated with FimV-GFP and GFP (-C). **F** UPLC chromatograms showing soluble PG fragments released by *E. coli* DH5α sacculus digestion with muramidase, and the fragments co-immunoprecipitated with FimV-GFP and GFP (-C). **G** Quantification of PG main features as indicated in Materials and Methods: total area below the chromatogram (left), crosslink (center) and average chain length (right). Bars represent the averages and standard error of the mean of three biological replicates. Student's t-test for unpaired samples not assuming equal variance was performed (ns: non-significant).

A

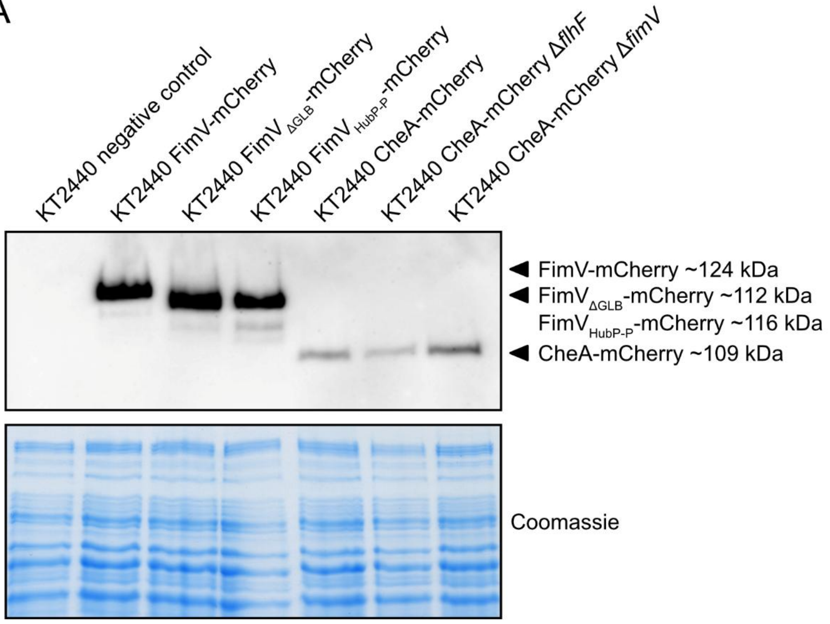

B

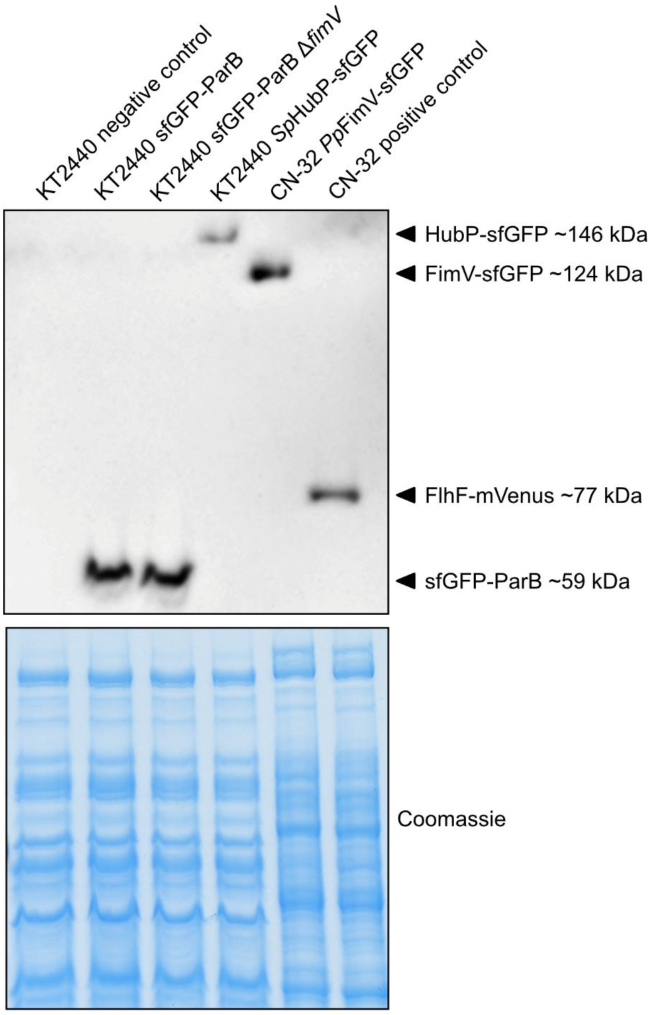

Supplementary Figure S3. Fusion proteins are stable produced in *P. putida* or *S. putrefaciens*. Protein samples from *Pseudomonas* or *Shewanella* were adjusted to an optical density of 10 and separated by SDS-PAGE. Afterwards the proteins were transferred to membranes by western blotting and visualized with primary antibodies against mCherry (**A**) or GFP (**B**). SDS-PAGE was stained with Coomassie as a loading control.

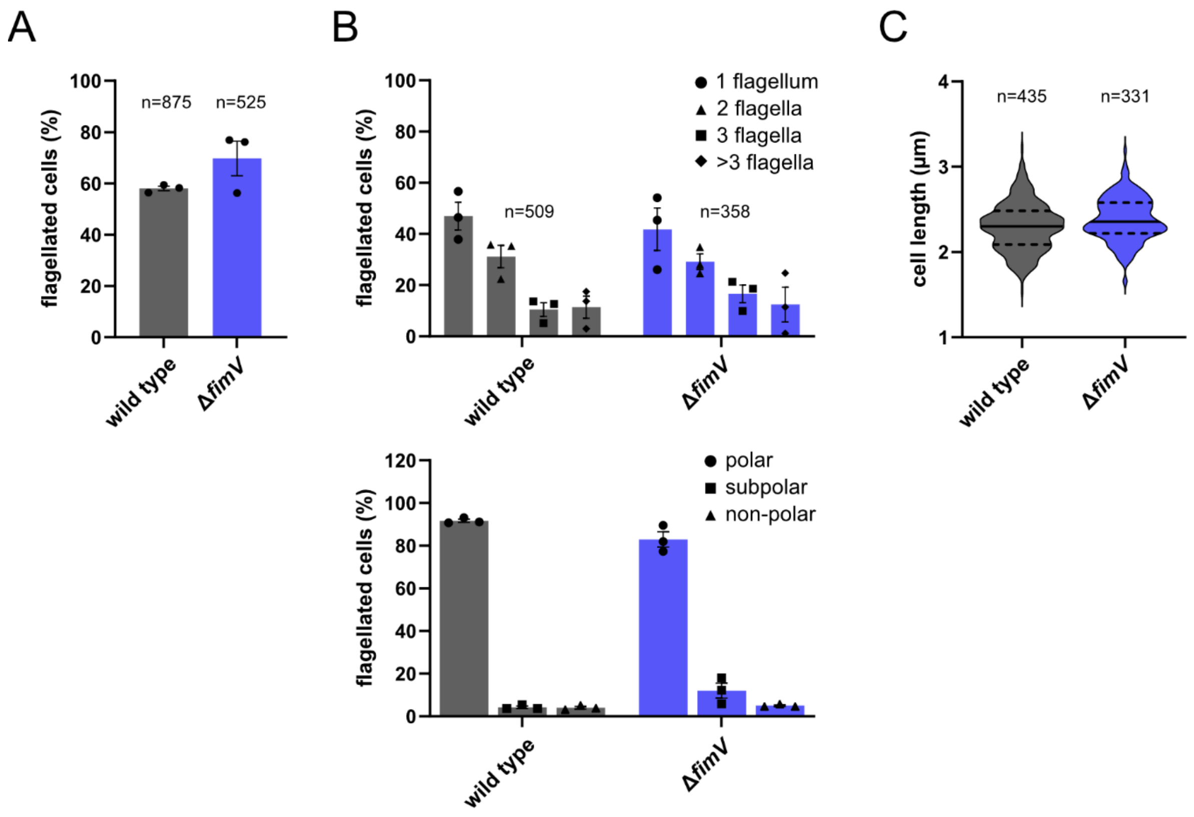

Supplementary Figure S4. Flagellation or cell length of *P. putida* is not altered by *fimV* deletion. *P. putida* cells harboring a serine to cysteine exchange at amino acid position 267 were stained with Alexa 488 maleimide dye (ThermoFisher) to visualize flagellar filaments. Data was analyzed as follows: **A** number of flagellated cells in a population, **B** the number of flagella per cell (top) and positioning of those flagella (bottom). Data from three independent experiments and corresponding error bars are shown. **C** The violin plot displays cell lengths measured using the Bacstalk software. The solid line indicates the median while the dashed lines represent the 25th and 75th percentiles (interquartile range). Total number of cells (n=) for each data analysis is indicated.

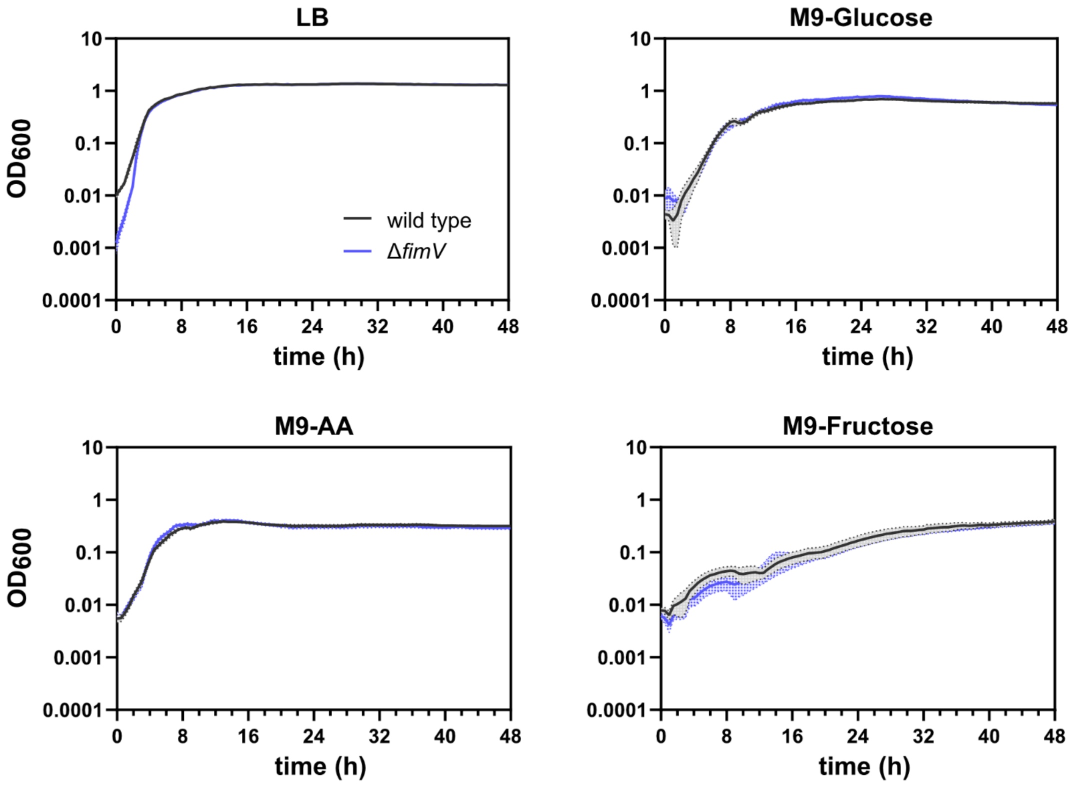

Supplementary Figure S5. Deletion of *fimV* does not influence the growth behavior. Growth (measured in OD_{600}) of the WT and *fimV* deletion mutants in LB and M9 medium, supplemented with different carbon sources: Glucose (0.4%), amino acids L-arginine and L-glutamine (M9-AA, 5 mM each), fructose (0.4%). Data from three independent experiments with standard error of mean are shown.

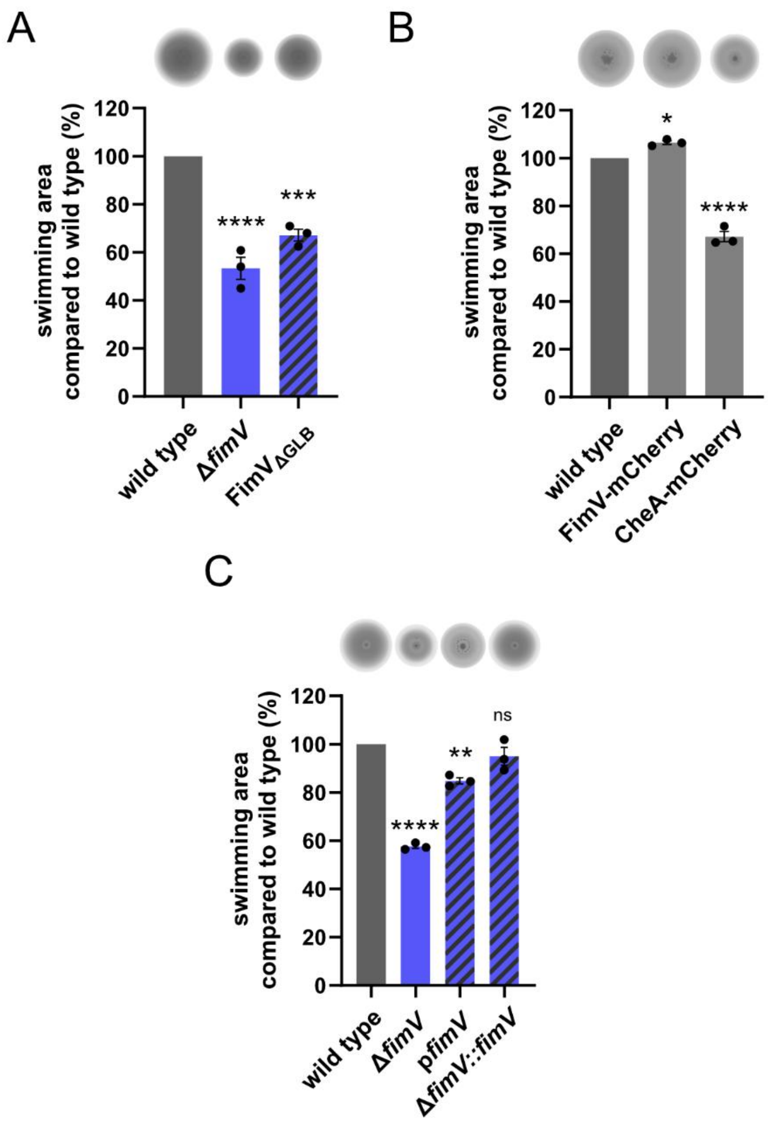

Supplementary Figure S6. Swimming behavior of *P. putida*. A Swimming behavior of *fimV* deletion or deletion of immunoglobulin-like domain (FimV$_{\Delta GLB}$) compared to that of the WT (set to 100%). Asterisks represent *p*<0.0001 and *p*=0.0004, respectively (comparison to the WT). **B** The FimV-mCherry fusion protein does not impair the swimming behavior while the CheA-mCherry fusion shows a slight reduction (*p*=0.0240 and *p*<0.0001). **C** Swimming abilities of plasmid-based complementation (p*fimV*, *p*=0.0017) and reintegration into native FimV position (*ΔfimV::fimV*, *p*=0.2476) of *fimV* deletion (*p*<0.0001) compared to the WT (set to 100%). Strains that were compared with each other were always spotted on the same plate. Data from three independent experiments with error bars are shown. For statistical analysis, one-way ANOVA was performed (ns: non-significant).

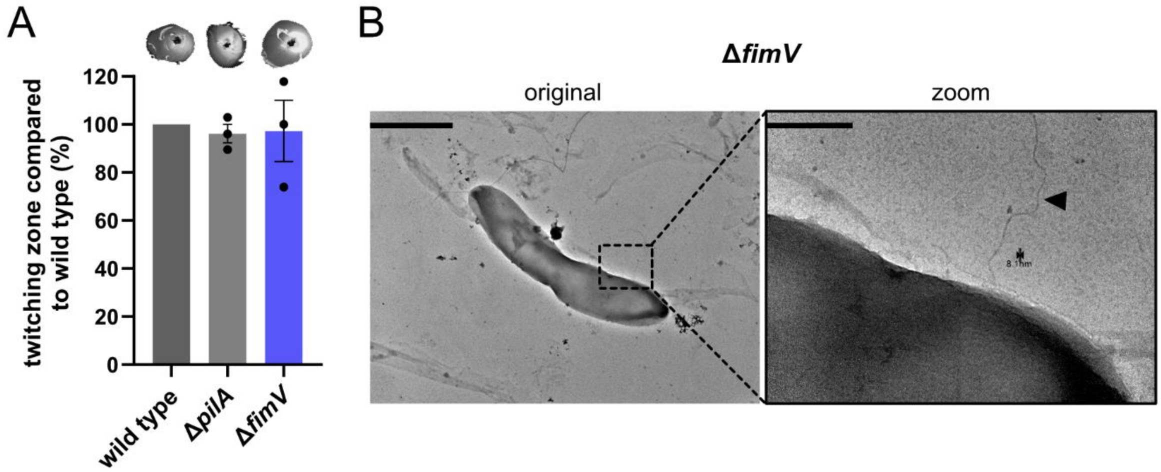

Supplementary Figure S7. Putative pilus-mediated twitching motility and pilus-like structure of *P. putida*. A Putative twitching behavior of *pilA* and *fimV* deletion mutant in comparison to WT (here *ΔfliC* to eliminate the influence of the flagellum) set to 100% between 1% LB agar and the bottom surface of the petri dish. Strains that were directly compared were always spotted on the same plate. Data from three independent experiments with error bars are shown. **B** Transmission electron photograph showing pilus-like structure (approximate size of 8 nm, indicated by the arrow) of *fimV* deletion mutant. Scale bar represents 2 µm (original) and 500 nm (zoom), respectively.

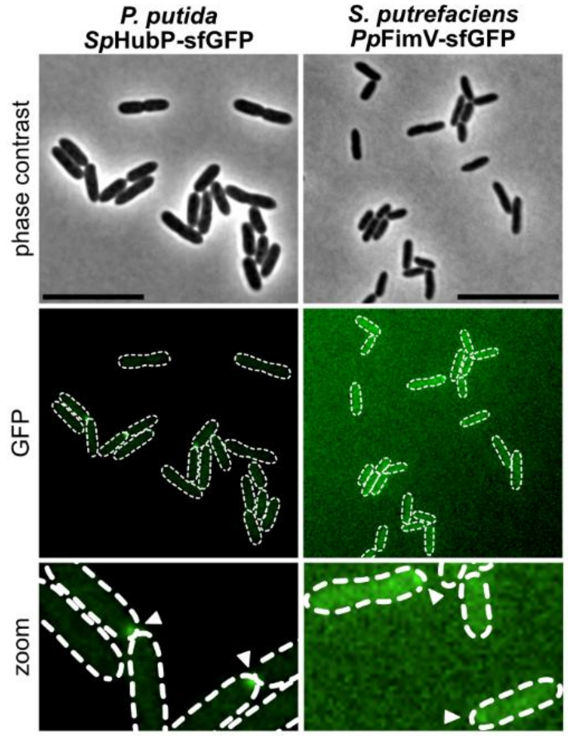

Supplementary Figure S8. Localization behavior of *Sp*HubP or *Pp*FimV in *P. putida* or *S. putrefaciens*, respectively. Microscopic pictures of *Sp*HubP in *P. putida* (left) or *Pp*FimV in *S. putrefaciens* (right) carrying translational fusions of the corresponding *Sp*HubP/*Pp*FimV proteins and sfGFP (green). Arrows indicate position of fluorescent *Sp*HubP/*Pp*FimV cluster. Scale bar equals 5 µm.
